# Supplementary material for: Targeting Mitochondrial COX-2 Enhances Chemosensitivity via Drp1-Dependent Remodeling of Mitochondrial Dynamics in Hepatocellular Carcinoma
Source: Cancers (Basel). 2022 Feb 6;14(3):821. doi: 10.3390/cancers14030821 (PMC8834292; doi:10.3390/cancers14030821)
Supplement: Supplementary file 1 [file cancers-14-00821-s001.zip › cancers-1547693-supplementary.pdf]

Supplementary Material: Targeting Mitochondrial COX-2 Enhances Chemosensitivity via Drp1-Dependent Remodeling of Mitochondrial Dynamics in Hepatocellular Carcinoma

Lin Che [1], Jia-Shen Wu, Ze-Bang Du, Yu-Qiao He, Lei Yang, Jin-Xian Lin, Zhao Lei, Xiao-Xuan Chen, Dong-Bei Guo, Wen-Gang Li, Yu-Chun Lin, and Zhong-Ning Lin

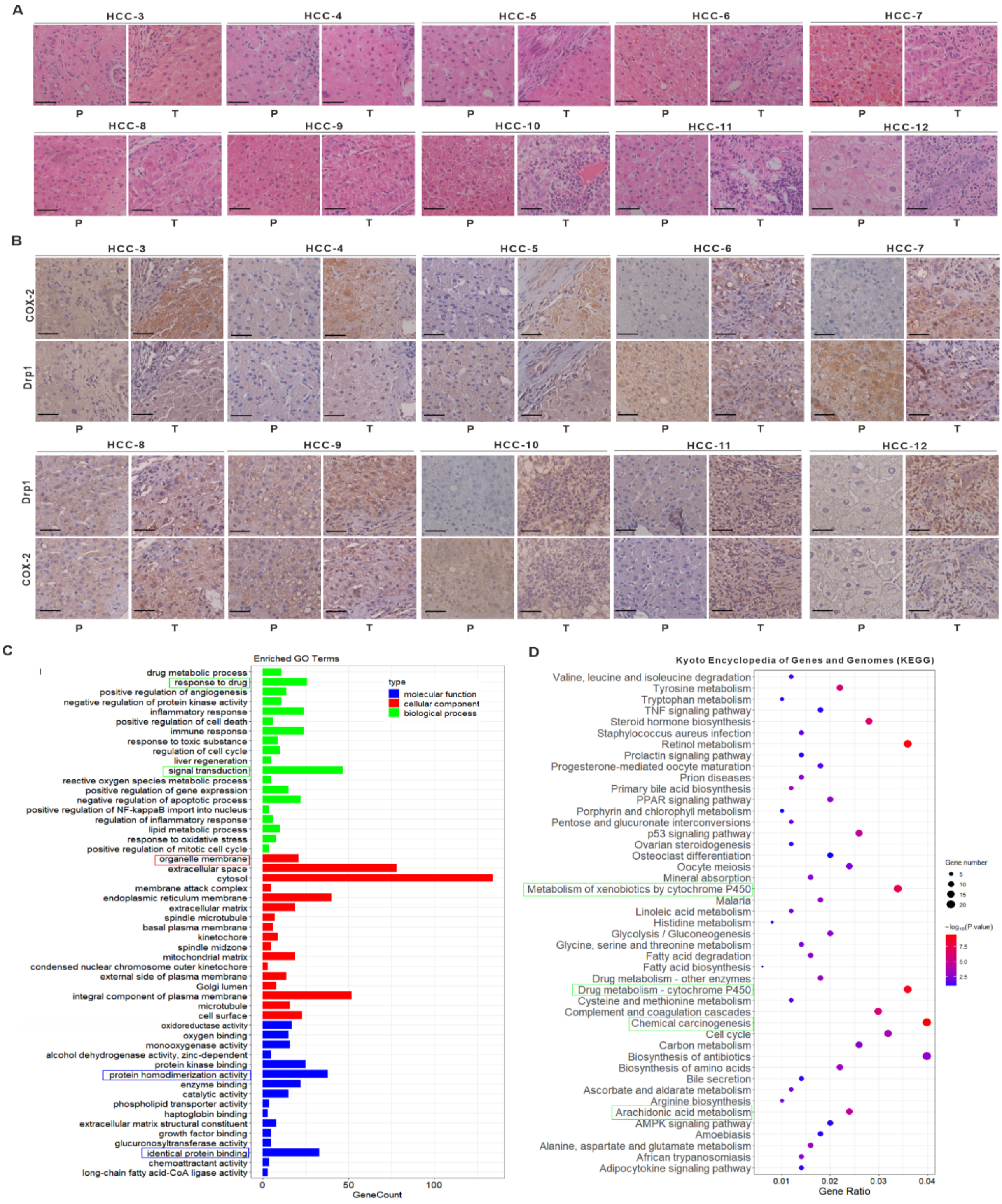

Figure S1. Upregulation of COX-2 and Drp1 is associated with poorer prognosis of HCC patients. (**A**–**B**) Twelve paired tumor tissues and peritumor tissues from HCC patients were collected and analyzed. (**A**) Representative hematoxylins and eosin (HE) staining images of the paired peritumor (P) and tumor (T) tissues from the HCC samples of #3–#12. Scale bar, 50 μm. (**B**) Representative IHC staining images for COX-2 and Drp1 expression in the paired peritumor (P) and tumor (T) tissues from the HCC samples of #3–#12 were shown. Scale bar, 50 μm. (**C,D**) The differentially expressed genes (DEGs), including *PTGS2* genes, were identified from a public RNA-seq dataset (Accession No.: GSE104310). (**C**) Gene ontology (GO) enrichment analysis of DEGs was used to identify pathways that was significantly affected by the differential expression of *PTGS2* in HCC patients. (**D**) KEGG pathway analysis of DEGs in HCC samples. COX-2 related pathways enriched significantly were shown.

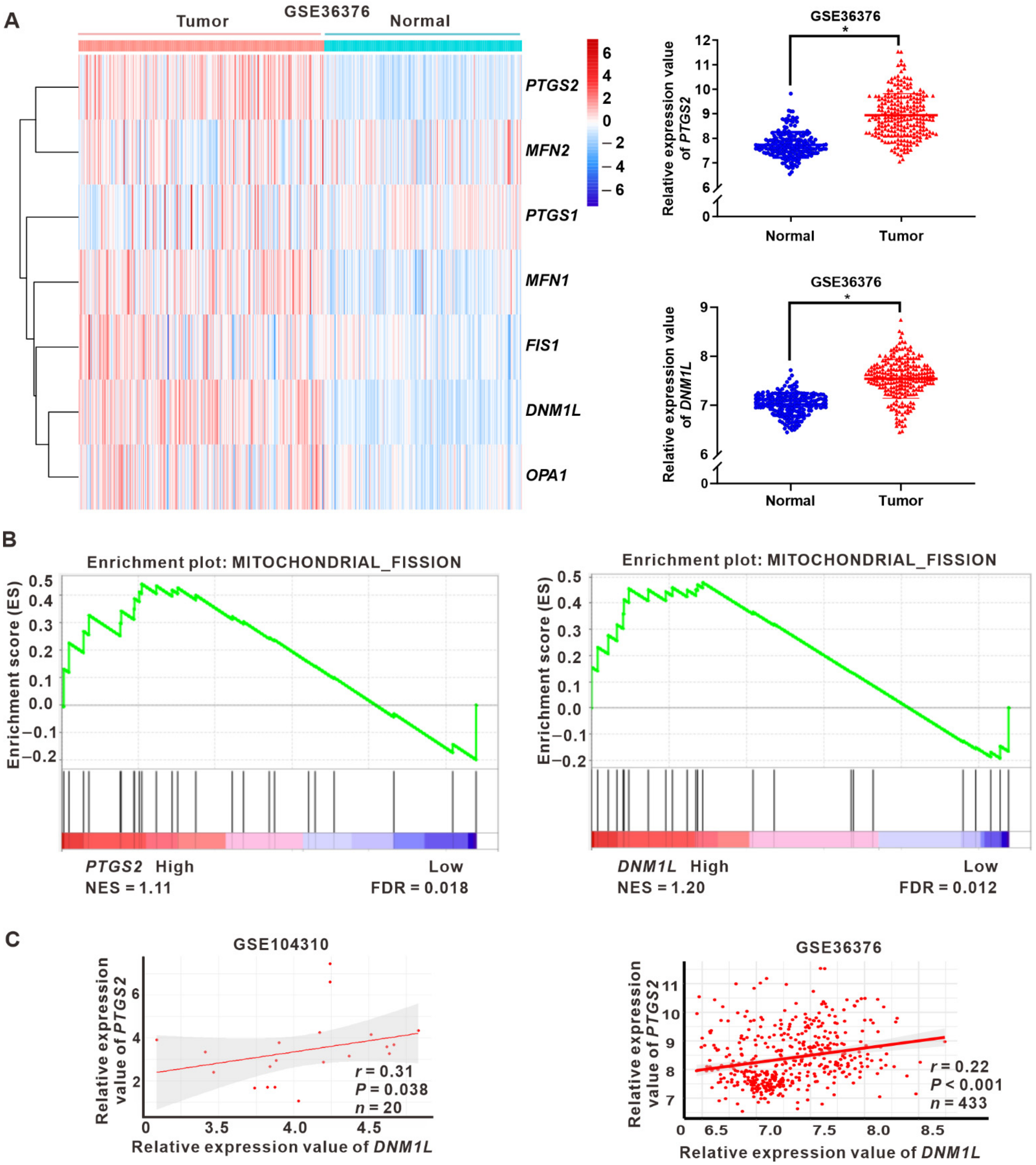

Figure S2. Upregulation of relative expression levels of *PTGS2* and *DNM1L* genes in HCC patients. (**A**–**C**) Gene expression analysis of RNA-seq dataset (GSE36376) in tumor and normal tissues samples from HCC patients (n = 433). (**A**) Heatmap for the relative expression levels of mitochondria-related DEGs (Left). The significant increases of *PTGS2* (Upper) and *DNM1L* (Below) in HCC tumor samples were shown. * $p < 0.05$, compared to the normal group. (**B**) GSEA indicated significant correlations between *PTGS2*, *DNM1L* genes expression and mitochondrial fission signatures. (**C**) The correlation between the expression of *PTGS2* and *DNM1L* was calculated by Pearson's correlation analysis.

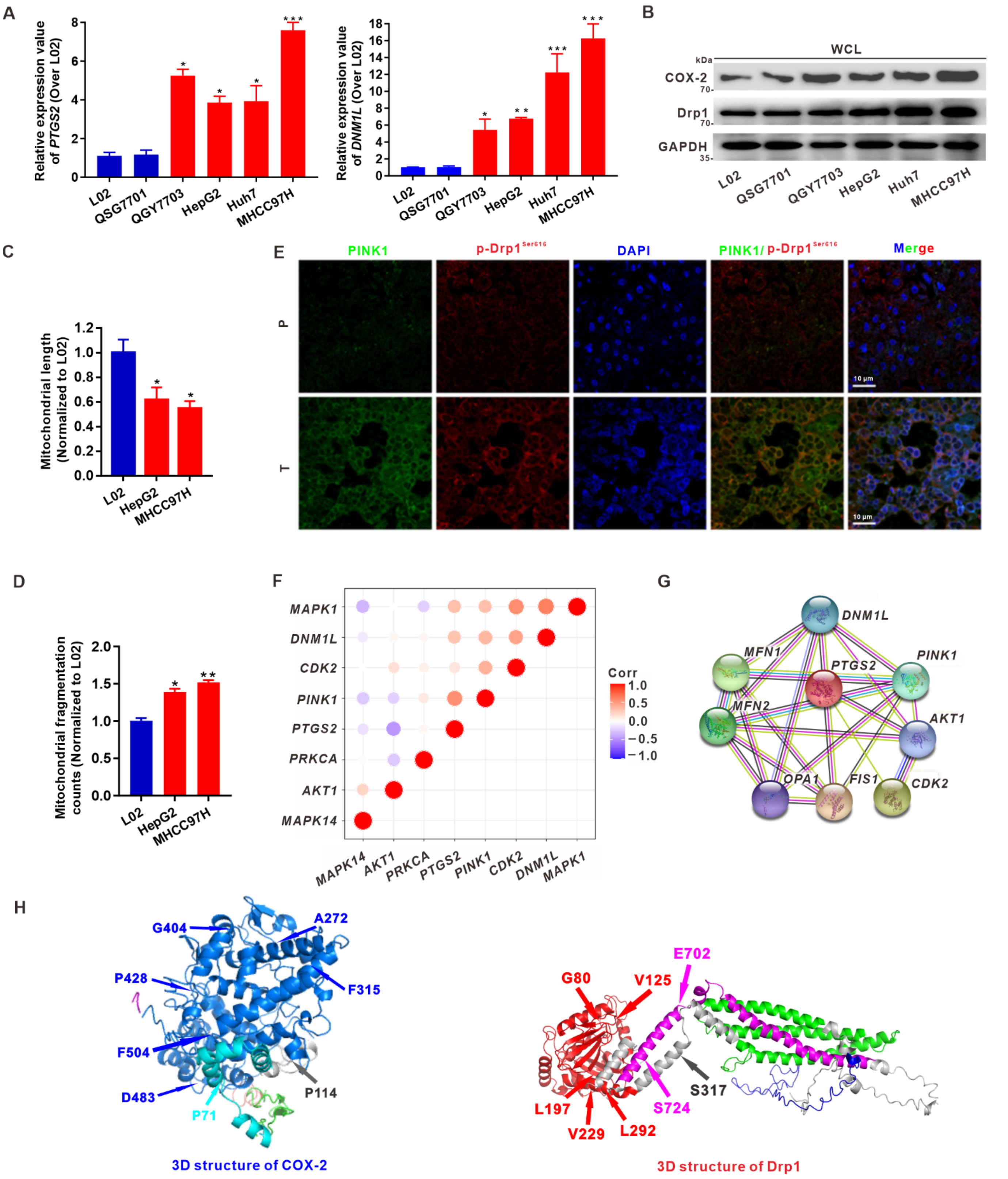

Figure S3. Activation of p-Drp1^{Ser616} enhances mitochondrial fission and its interaction with COX-2 in HCC cells. (**A**,**B**) Four HCC cell lines (QGY7703, HepG2, Huh7, and MHCC97H) and two normal hepatic cell lines (L02 and QSG7701) were used to screen. (**A**) Levels of *PTGS2* and *DNM1L* mRNA were detected by qRT-PCR. Data are expressed as mean ± SD. * $p < 0.05$, ** $p < 0.01$, *** $p < 0.001$, compared to L02 cells. (**B**) Whole-cell lysates (WCL) were subjected to detect the levels of COX-2 and Drp1 by western blot. Full Western Blot images can be found in Figures S9 and S10. (**C**) Quantitative analysis of mitochondrial length in transmission electron microscopy (TEM) imaging is

shown in bar graphs. Data are shown as mean ± SD. * $p < 0.05$, compared to L02 cells. (**D**) Quantification of mitochondrial fragmentation counts in IF imaging is shown in bar graphs. Data are shown as mean ± SD. * $p < 0.05$, compared to L02 cells. (**E**) Serial sections of the paired peritumor (P) and tumor (T) tissues were subjected to immunofluorescence analysis for evaluating the co-expression of PINK1 (green) and p-Drp1^{Ser616} (red), and the co-localization (yellow) between PINK1 and p-Drp1^{Ser616}. Scale bars, 10 µm. (**F**) The correlation between the expression of indicated genes, including *PINK1* and *PTGS2* or *DNM1L*, was analyzed with the GEO dataset (Accession No.: GSE49515). (**G**) Construction of protein-protein interaction (PPI) network with mitochondria-related genes. The potential direct interaction between COX-2 and Drp1, encoded by *PTGS2* and *DNM1L* genes, respectively. (**H**) Predicted 3D structures of human COX-2 protein (Left) and Drp1 protein (Right) using I-TASSER.

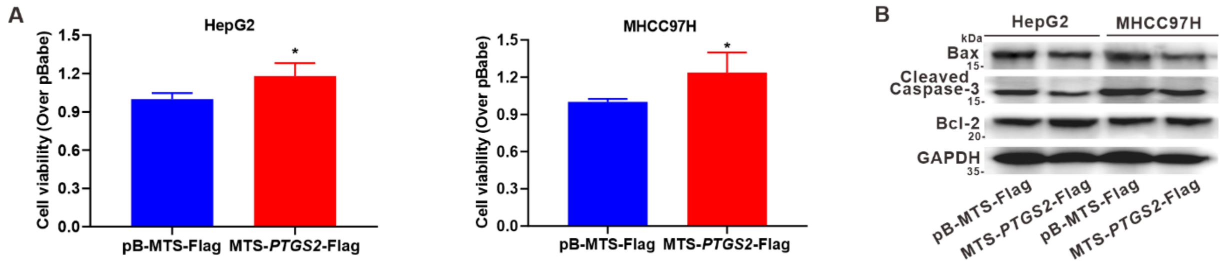

Figure S4. Mito-COX-2 modulates mitochondrial fission by stabilizing the activity of p-Drp1^{Ser616} in HCC cells. (**A**,**B**) HepG2- and MHCC97H-MTS-*PTGS2*-Flag cells overexpressing mito-COX-2 were established, together with HepG2- and MHCC97H-pBabe control cells. (**A**) Cell viability was performed by MTS assays. * $p < 0.05$, compared to the corresponding control cells. (**B**) Levels of apoptosis related proteins Bax, Cleaved Caspase-3, and Bcl-2 were determined by western blot. Full Western Blot images can be found in Figures S9 and S10.

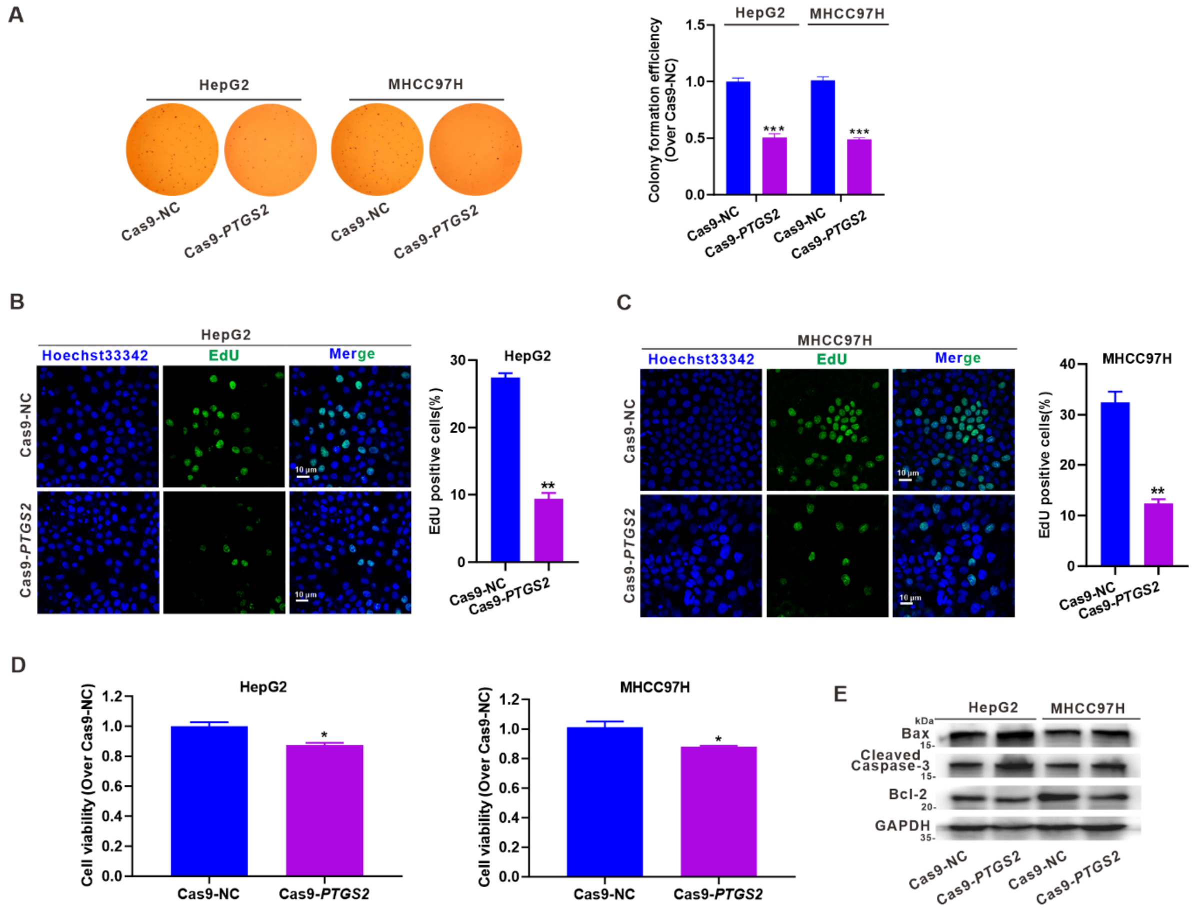

Figure S5. Suppression of mito-COX-2 translocation decreases its interaction with p-Drp1^{Ser616} and modulates mitochondrial fission in HCC cells. (**A**,**E**) HepG2- and MHCC97H-Cas9-*PTGS2* cells with

COX-2-knockdown were established, together with HepG2- and MHCC97H-Cas9-NC control cells. (**A**) The self-renewal capacity of the cells was measured by colony formation assay. Representative colony formation images (Left) and the efficiency of colony formation (Right) were shown. (**B**,**C**) Cell proliferation was evaluated by EdU incorporation assay (Left). Scale bar, 10 μm. Quantification of EdU positive cells ratio is shown in the bar graph (Right). (**D**) Cell viability was performed by MTS assays. Data are expressed as mean ± SD. * $p < 0.05$, ** $p < 0.01$, compared to the corresponding Cas9-NC control cells. (**E**) Levels of apoptosis related proteins Bax, Cleaved Caspase-3, and Bcl-2 were determined by western blot. Full Western Blot images can be found in Figures S9 and S10.

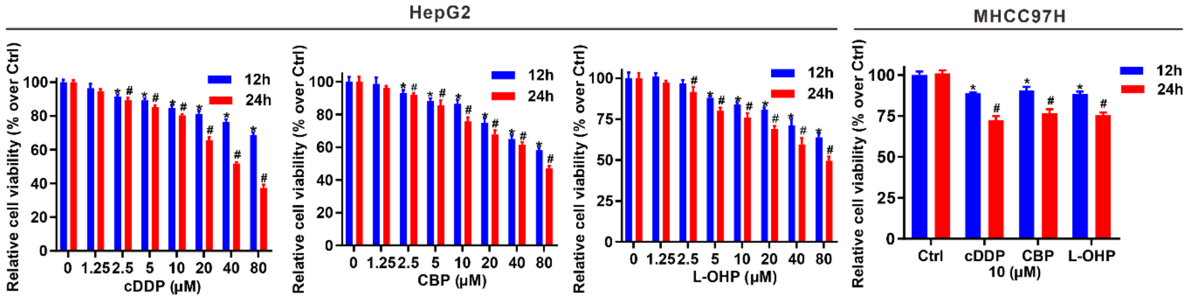

Figure S6. Mitochondrial translocation of COX-2 and mitochondrial fission related to the anti-cancer effect in platinum drug-treated HCC cells. Three commonly used platinum chemotherapy drugs, cisplatin (cDDP), carboplatin (CBP), and oxaliplatin (L-OHP) were administered in HCC cells. HepG2 and MHCC97H cells were treated with indicated concentrations of platinum drugs for 12 h or 24 h, and cell viability was performed by MTS assays. * $p < 0.05$, $^{\#}p < 0.05$, compared to the corresponding control groups.

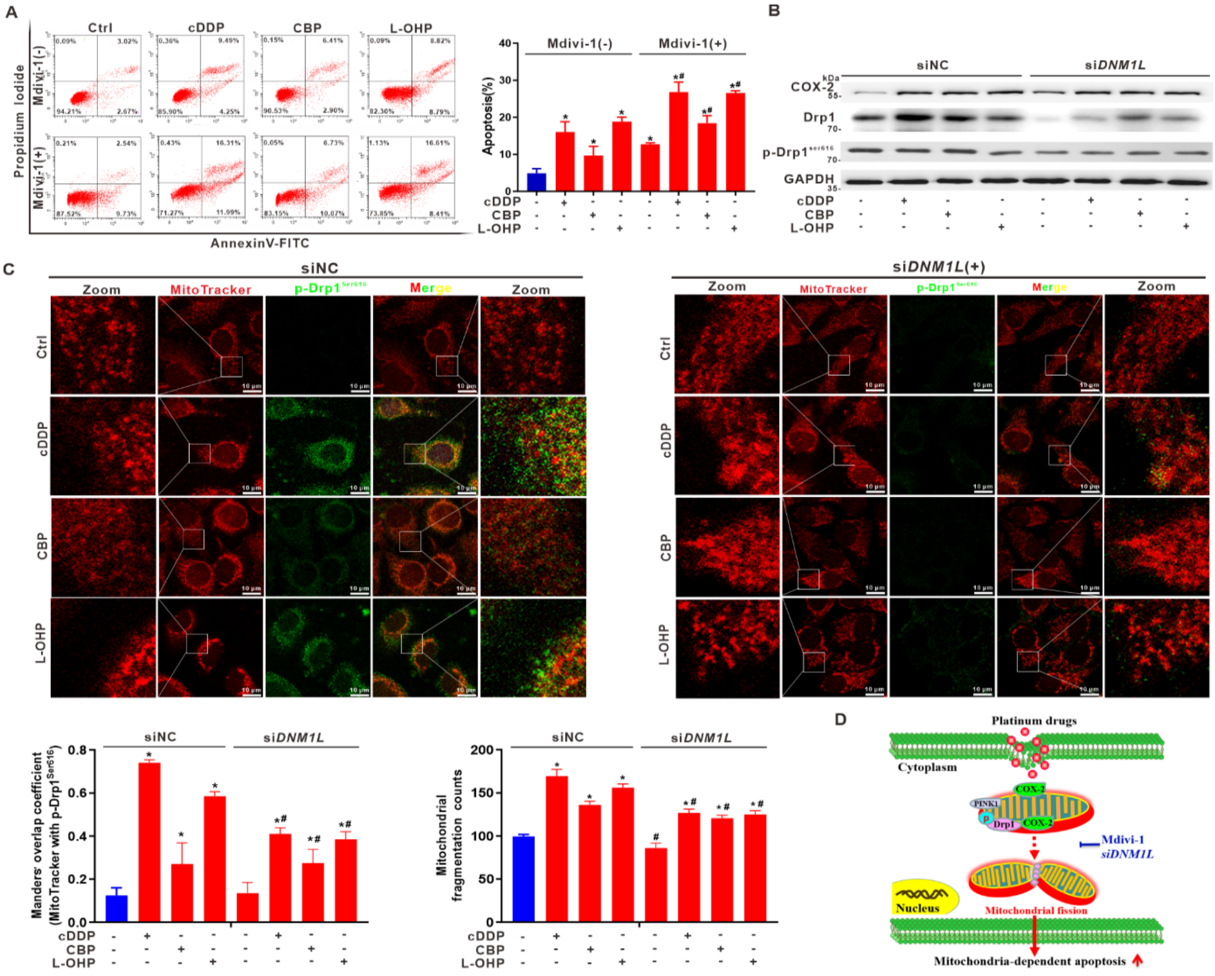

Figure S7. Suppression of Drp1 promotes apoptosis via inhibition of mito-COX-2/p-Drp1^{Ser616} interaction in platinum drug-treated HCC cells. Three commonly used platinum chemotherapy drugs, cisplatin (cDDP), carboplatin (CBP), and oxaliplatin (L-OHP) were administered on HCC cells. (**A**) HepG2 cells were pre-treated with or without Mdivi-1 (20 μM) for 12 h, followed by platinum drugs treatment for 12 h. Apoptosis rate was detected by staining with Annexin V-FITC/PI. The representative analysis of Flow cytometry was shown (Left). The quantification of apoptotic cells is shown in the bar graph (Right). * $p < 0.05$, compared to the corresponding control groups. $^{\#}$ $p < 0.05$, compared to the corresponding Mdivi-1(-) groups. (**B**,**C**) HepG2 cells were pre-treated with or without si*DNM1L* (50 nM) for 12 h, followed by platinum drugs treatment for 12 h. (**B**) Whole-cell lysates were subjected to western blot. Full Western Blot images can be found in Figures S9 and S10. (**C**) Representative images of mitochondria (red), p-Drp1^{Ser616} (green), and their colocalization (yellow) were shown in confocal microscopy (Upper). Scale bars: 10 μm. Manders' overlap coefficients for co-localization of p-Drp1^{Ser616} with mitochondria (Lower left) and mitochondrial fragmentation counts (Lower right) were calculated using IPP 6.0, while the quantification is shown in the bar graph. (**D**) Schematic representation showed the role of targeting inhibition of Drp1 suppressed the mito-COX-2/p-Drp1^{Ser616} interaction and its driven mitochondrial fission, and potentiated the pro-apoptotic effect and anti-tumor sensitivity induce by platinum drugs in HCC cells.

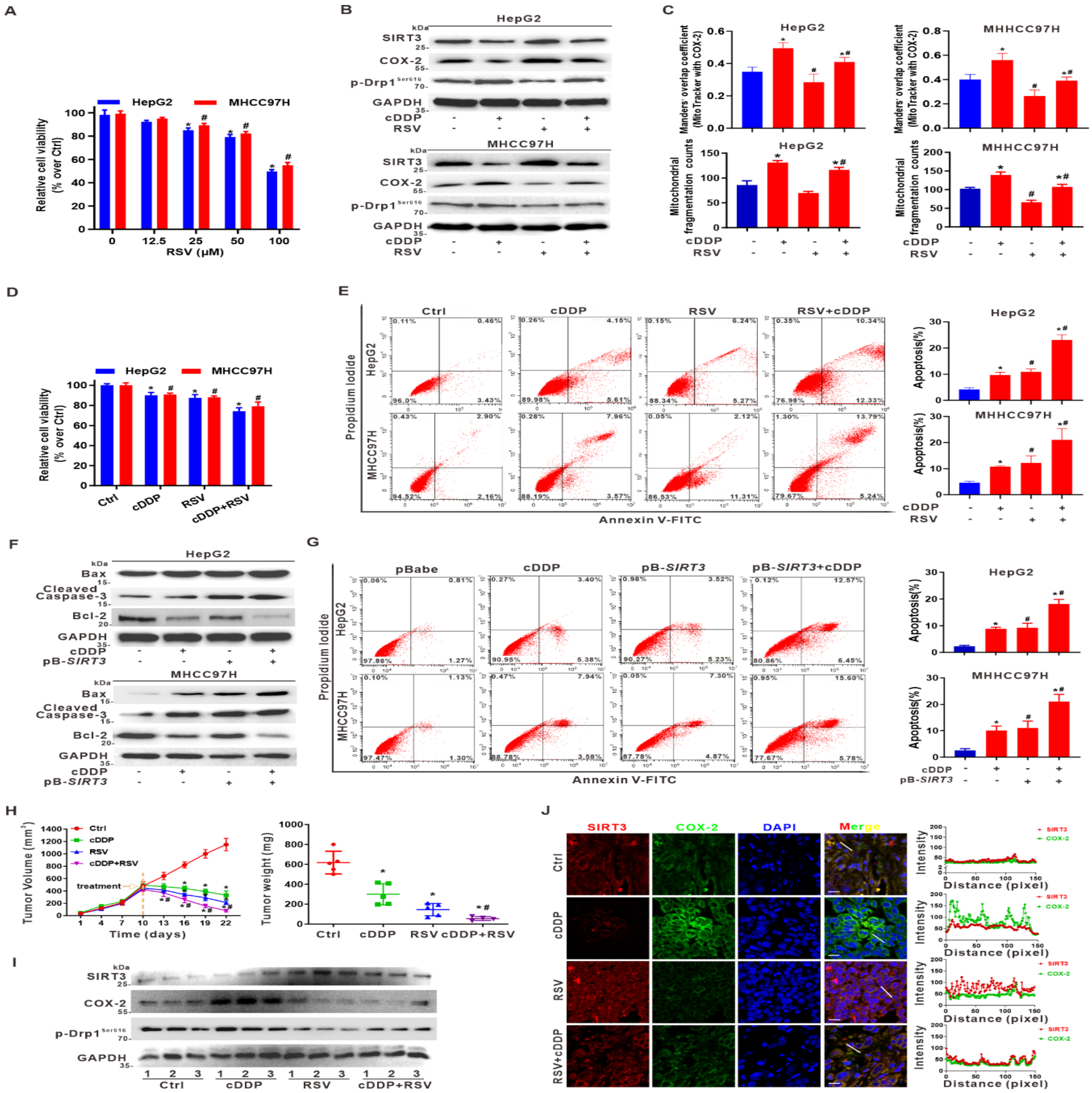

Figure S8. Deacetylation of mito-COX-2 via SIRT3 activation mediates higher sensitivity of HCC to cisplatin by inhibiting mito-COX-2/p-Drp1^{Ser616} interactions in vitro and in vivo. (**A**–**E**) In vitro study, HepG2 and MHCC97H cells were pre-treated with or without resveratrol (RSV, 50 μM) for 24 h, followed by cDDP treatment for 12 h. (**A**) HepG2 and MHCC97H cells were treated with indicated concentrations of RSV for 24 h, and cell viability was performed by MTS assays. $^{*,\#}$ $p < 0.05$, compared to the corresponding control (Ctrl) groups. (**B**) The expression of SIRT3, COX-2, and p-Drp1^{Ser616} was determined by western blot. Full Western Blot images can be found in Figures S9 and S10. (**C**) Immunofluorescence staining for mitochondria and COX-2 was captured by confocal microscopy. Manders' overlap coefficients for co-localization of COX-2 with mitochondria (Upper) and mitochondrial fragmentation counts (Lower) were calculated using IPP 6.0, while the quantification is shown in bar graph. * $p < 0.05$, compared to the corresponding control groups. $^{\#}$ $p < 0.05$, compared to cDDP-treated alone group. (**D**) Cells viability was measured with the MTS assay. $^{*,\#}$ $p < 0.05$, compared to the corresponding control (Ctrl) groups. (**E**) Apoptosis rate was detected by Annexin V-FITC/PI staining. Representative analysis of Flow cytometry was shown (Left). The quantification of apoptotic cells is shown in the bar graph (Right). * $p < 0.05$, compared to the corresponding control groups. $^{\#}$ $p < 0.05$, compared to cDDP-treated alone group. (**F**–**G**) SIRT3-overexpressing HepG2- and MHCC97H-*SIRT3*-Flag cells were constructed, together with HepG2- and MHCC97H-pBabe-Flag control cells. (**F**) The levels of apoptosis related proteins Bax, cleaved Caspase-3, and Bcl-2 were determined by western blot. Full Western Blot images can be found in Figures S9 and S10. (**G**) Apoptosis rate was detected by Annexin V-FITC/PI staining. Representative analysis of Flow cytometry was shown (Left). The quantification of apoptotic cells is shown in the bar graph (Right). * $p < 0.05$, compared to pBabe control group. $^{\#}$ $p < 0.05$, compared to cDDP-treated alone group. (**H**–**J**) In vivo study, BALB/c nude mice bearing HepG2 cells as primary xenografts were randomly assigned to four experimental groups ($n = 5$ for each group) at day 10. Subcutaneous injection was conducted for treatment with normal saline (Ctrl), cDDP (1 mg/kg), RSV (50 mg/kg), or a combination of cDDP and RSV every three days, for four times. (**H**) Tumor growth curves reported the xenograft tumor volume (Left), and the weights of xenograft tumors were shown (Right). * $p < 0.05$, compared to control (Ctrl) group. $^{\#}$ $p < 0.05$, compared to cDDP-treated alone group. (**I**) Levels of SIRT3, COX-2, and p-Drp1^{Ser616} in representative xenograft tumor tissues were determined by western blot. Full Western Blot images can be found in Figures S9 and S10. (**J**) Serial section of xenograft tumors was subjected to immunofluorescence (IF) assay to evaluate the expression of SIRT3 (red), and COX-2 (green), while the colocalization was shown in Merge (Left). Fluorescence curves were generated using Zen 2010 software (Right). Scale bars, 10 μm.

Fig.1

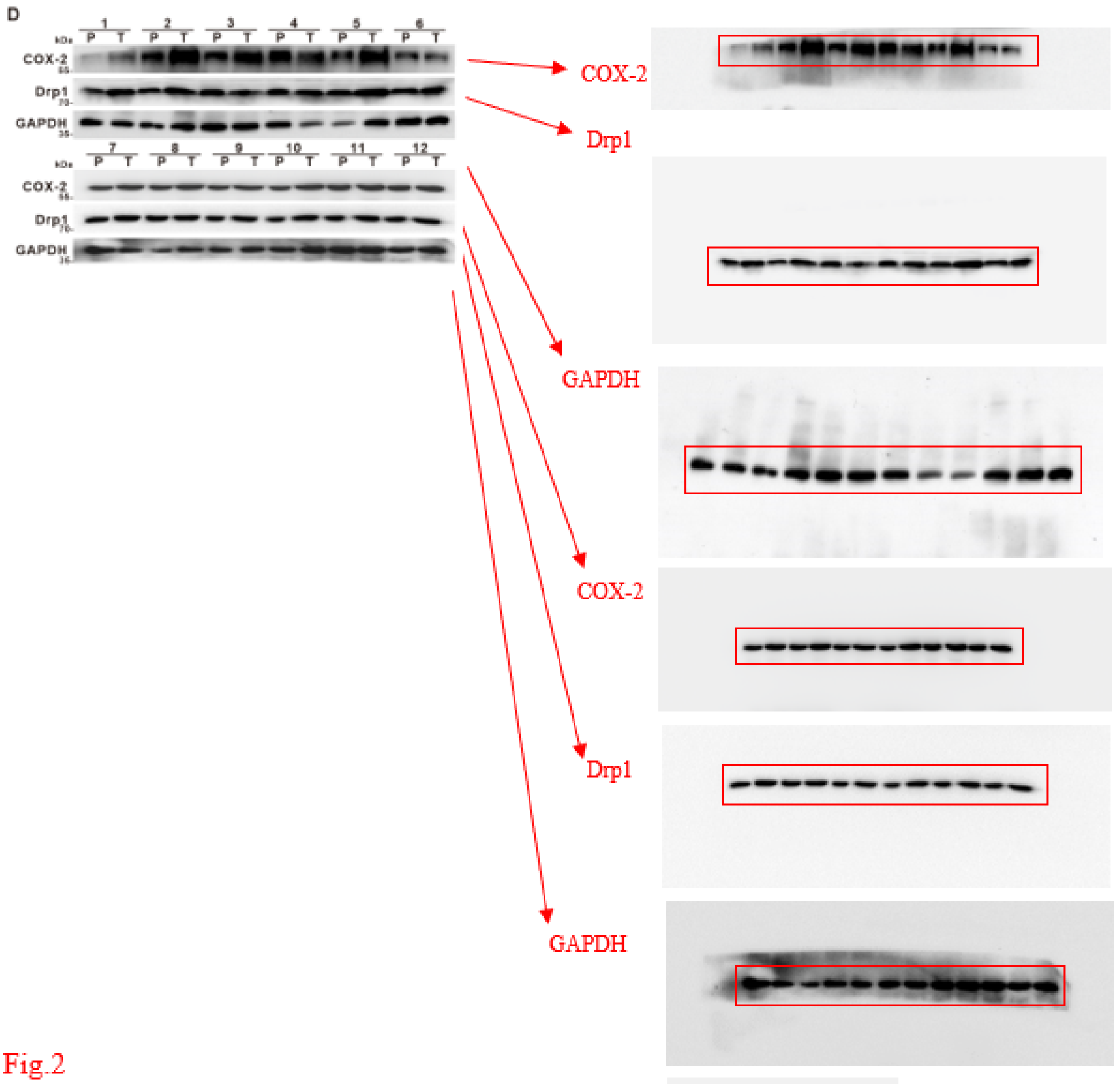
D
1 2 3 4 5 6
P T P T P T P T P T P T
COX-2
Drp1
GAPDH
7 8 9 10 11 12
P T P T P T P T P T P T
COX-2
Drp1
GAPDH
COX-2
Drp1
GAPDH
COX-2
Drp1
GAPDH

Fig.2

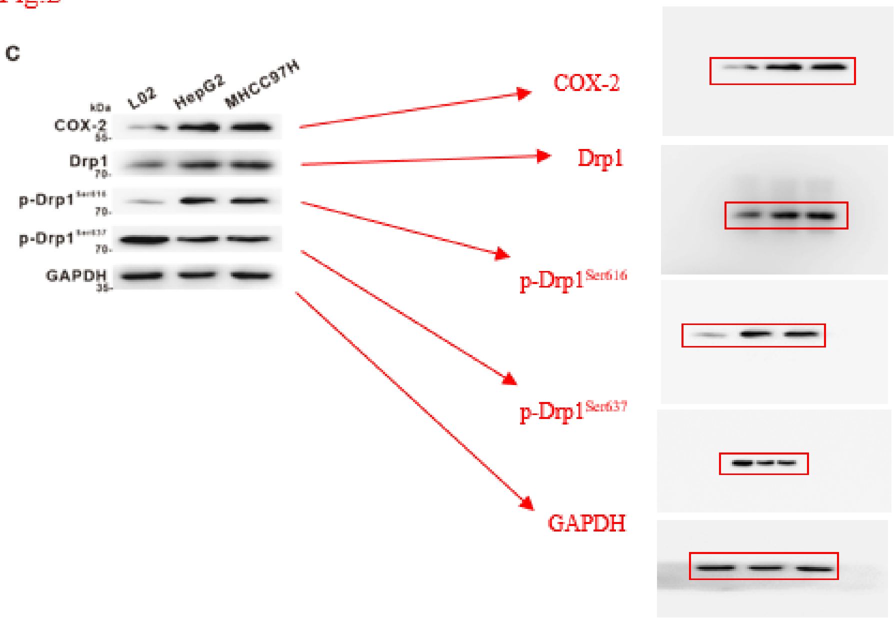
C
kDa
L02
HepG2
MHCC97H
COX-2
Drp1
p-Drp1 Ser616
p-Drp1 Ser637
GAPDH
COX-2
Drp1
p-Drp1 Ser616
p-Drp1 Ser637
GAPDH

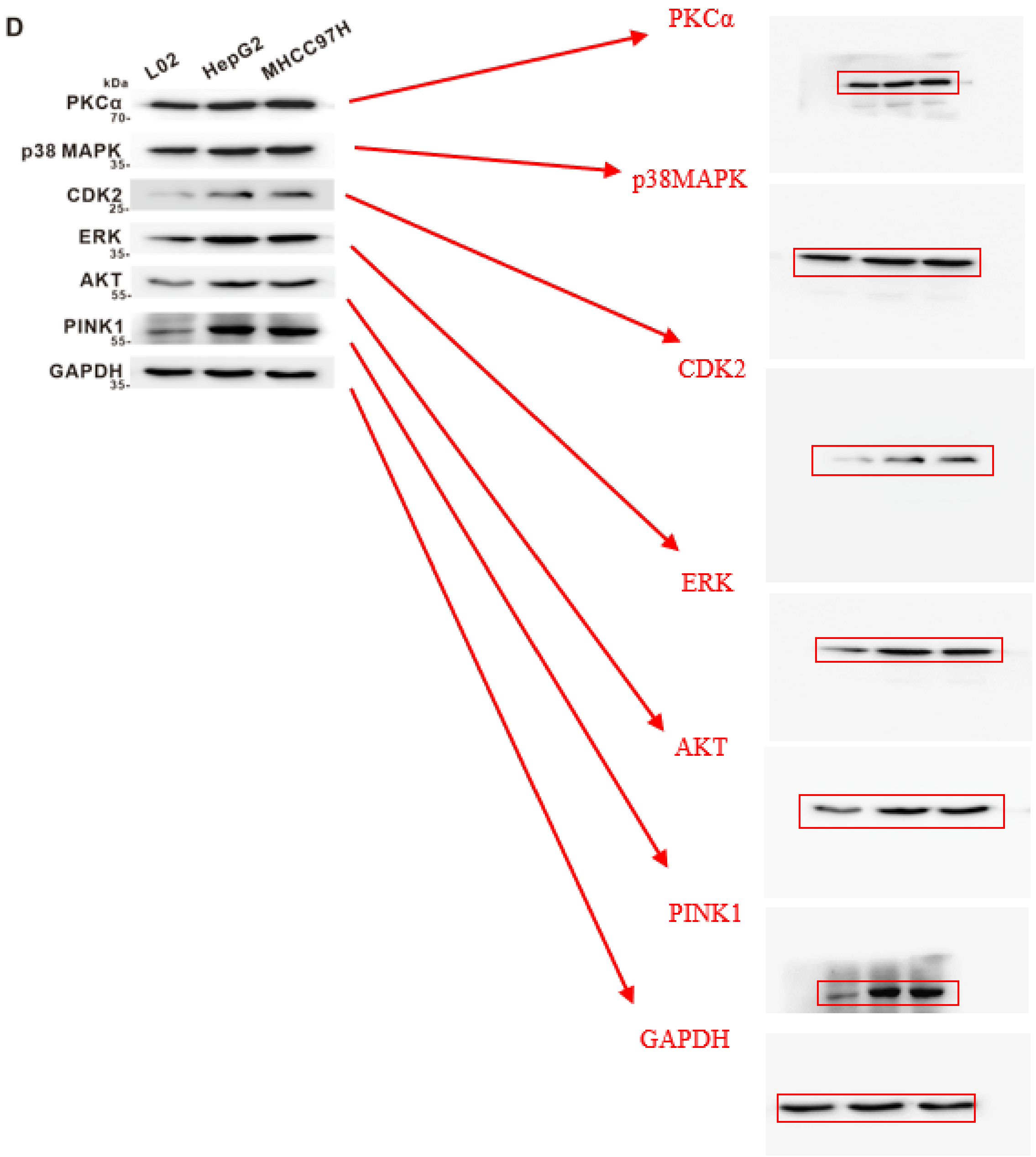
D
kDa
L02
HepG2
MHCC97H
PKCα
70-
p38 MAPK
35-
CDK2
25-
ERK
35-
AKT
55-
PINK1
55-
GAPDH
35-
PKCα
p38MAPK
CDK2
ERK
AKT
PINK1
GAPDH

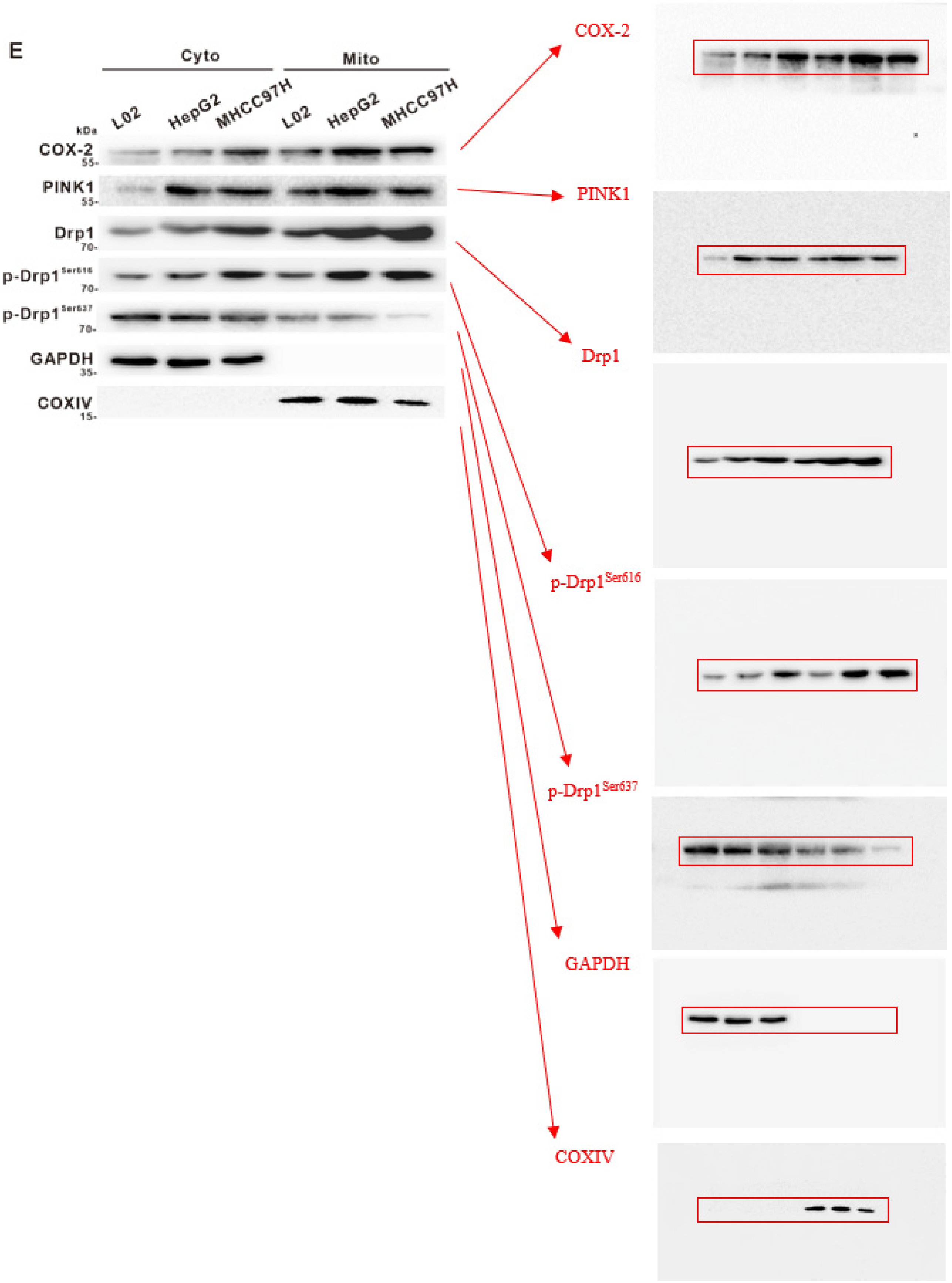
E
Cyto
Mito
L02
HepG2
MHCC97H
L02
HepG2
MHCC97H
kDa
COX-2
55-
PINK1
55-
Drp1
70-
p-Drp1 Ser616
70-
p-Drp1 Ser637
70-
GAPDH
35-
COXIV
15-
COX-2
PINK1
Drp1
p-Drp1 Ser616
p-Drp1 Ser637
GAPDH
COXIV

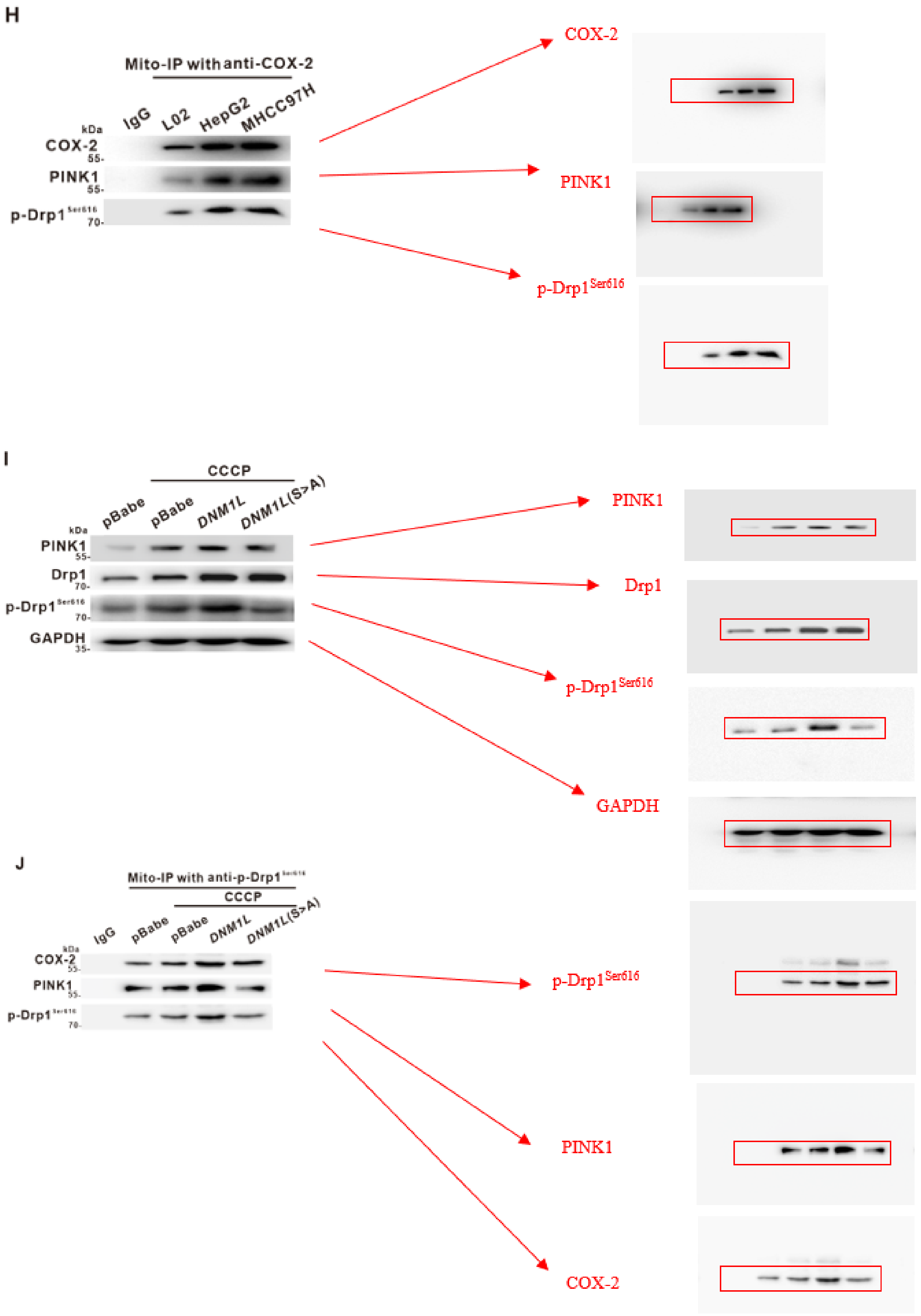
H
Mito-IP with anti-COX-2
kDa
IgG
L02
HepG2
MHCC97H
COX-2
55-
PINK1
55-
p-Drp1 Ser616
70-
COX-2
PINK1
p-Drp1 Ser616
I
CCCP
pBabe
pBabe
DNM1L
DNM1L(S>A)
kDa
PINK1
55-
Drp1
70-
p-Drp1 Ser616
70-
GAPDH
35-
PINK1
Drp1
p-Drp1 Ser616
GAPDH
J
Mito-IP with anti-p-Drp1 Ser616
CCCP
IgG
pBabe
pBabe
DNM1L
DNM1L(S>A)
kDa
COX-2
55-
PINK1
55-
p-Drp1 Ser616
70-
p-Drp1 Ser616
PINK1
COX-2

Fig.3

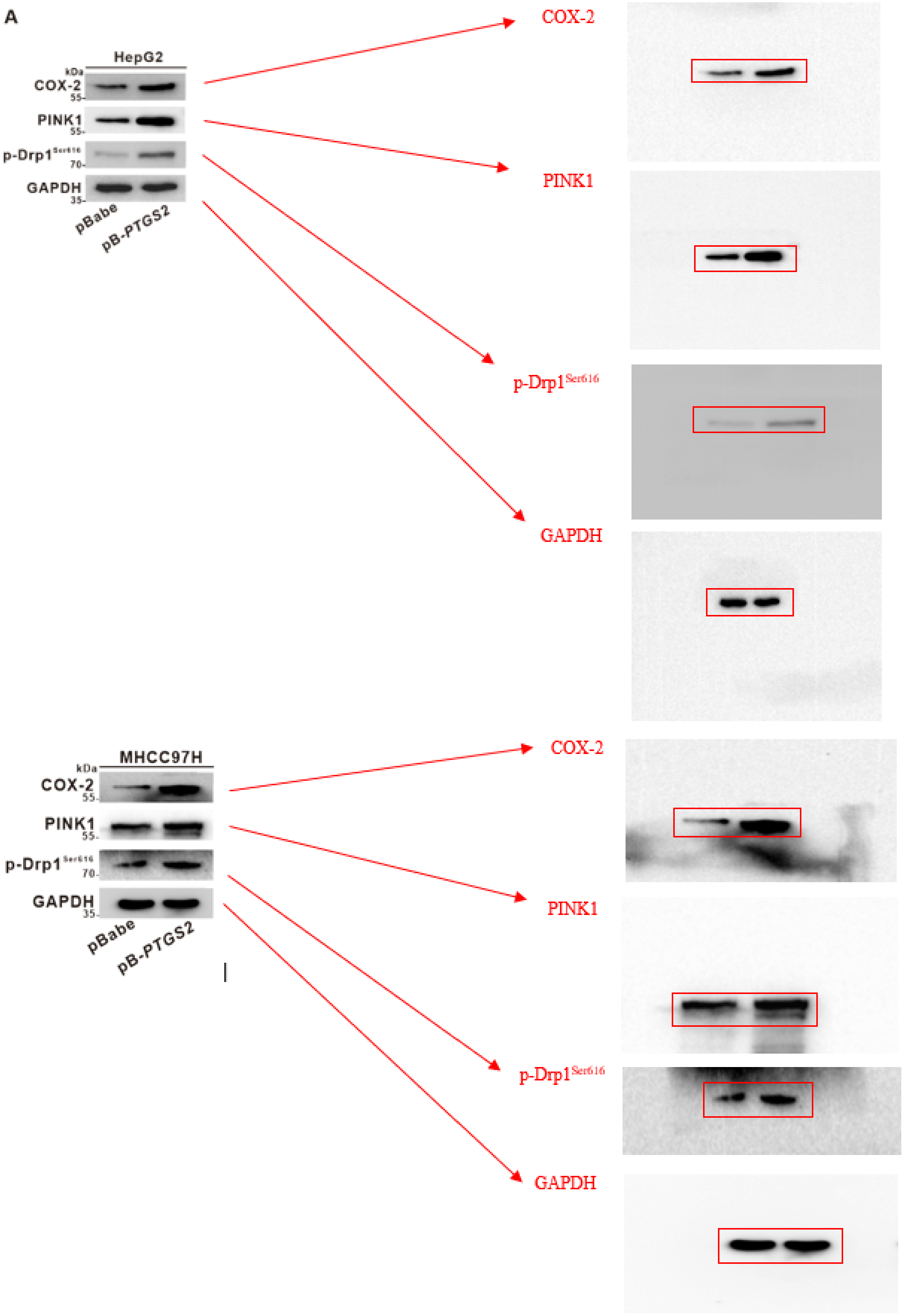
A
HepG2
kDa
COX-2
55
PINK1
55
p-Drp1 Ser616
70
GAPDH
35
pBabe
pB-PTGS2
COX-2
PINK1
p-Drp1 Ser616
GAPDH
MHCC97H
kDa
COX-2
55
PINK1
55
p-Drp1 Ser616
70
GAPDH
35
pBabe
pB-PTGS2
COX-2
PINK1
p-Drp1 Ser616
GAPDH

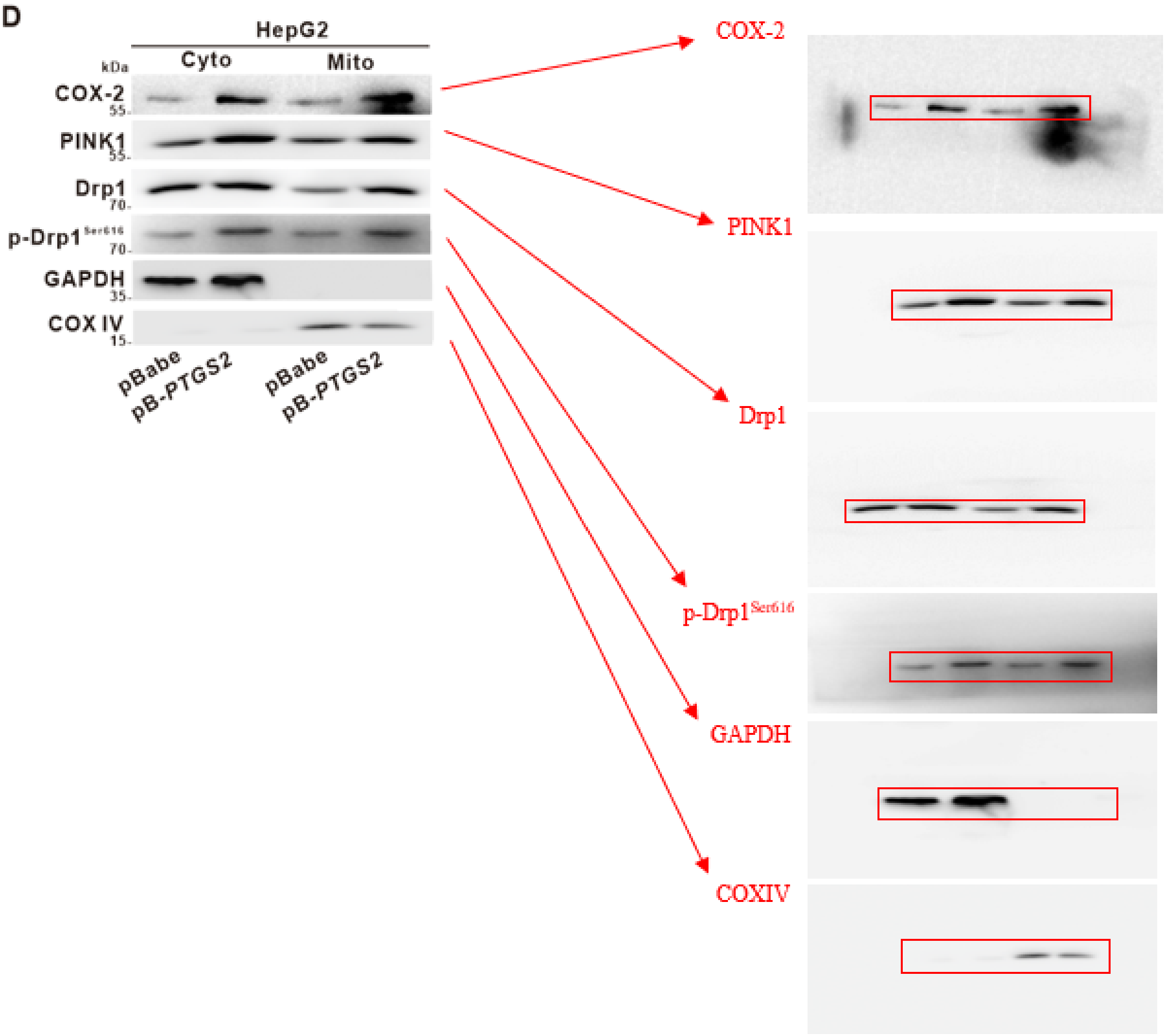
D
HepG2
Cyto
Mito
kDa
COX-2
PINK1
Drp1
p-Drp1 Ser616
GAPDH
COX IV
55
55
70
70
35
15
pBabe
pB-PTGS2
pBabe
pB-PTGS2
COX-2
PINK1
Drp1
p-Drp1Ser616
GAPDH
COXIV

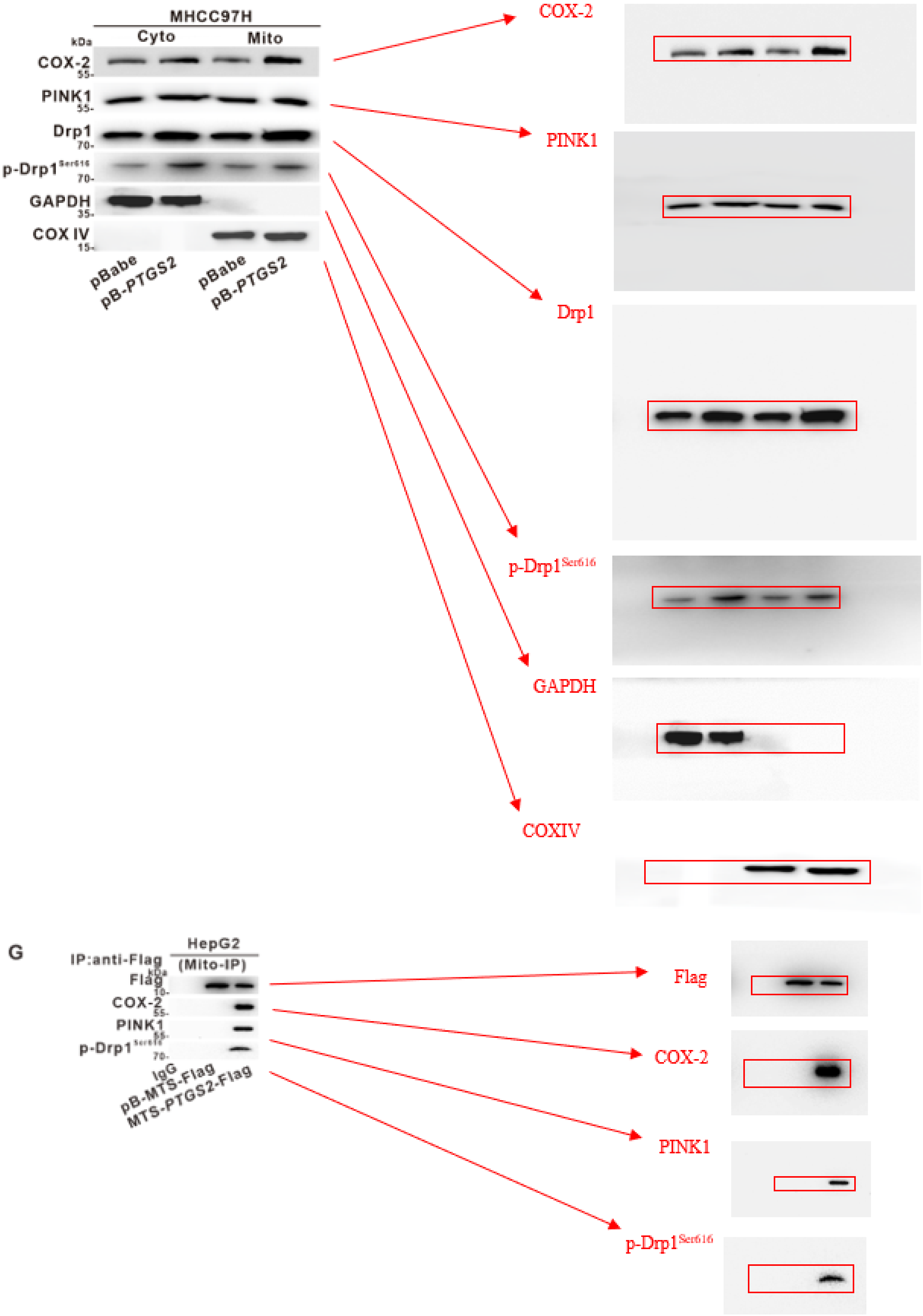
MHCC97H
Cyto
Mito
kDa
COX-2
55-
PINK1
55-
Drp1
70-
p-Drp1 Ser616
70-
GAPDH
35-
COX IV
15-
pBabe
pB-PTGS2
pBabe
pB-PTGS2
COX-2
PINK1
Drp1
p-Drp1 Ser616
GAPDH
COXIV
G
IP:anti-Flag
HepG2
(Mito-IP)
kDa
Flag
10-
COX-2
55-
PINK1
55-
p-Drp1 Ser616
70-
IgG
pB-MTS-Flag
MTS-PTGS2-Flag
Flag
COX-2
PINK1
p-Drp1 Ser616

Fig.4

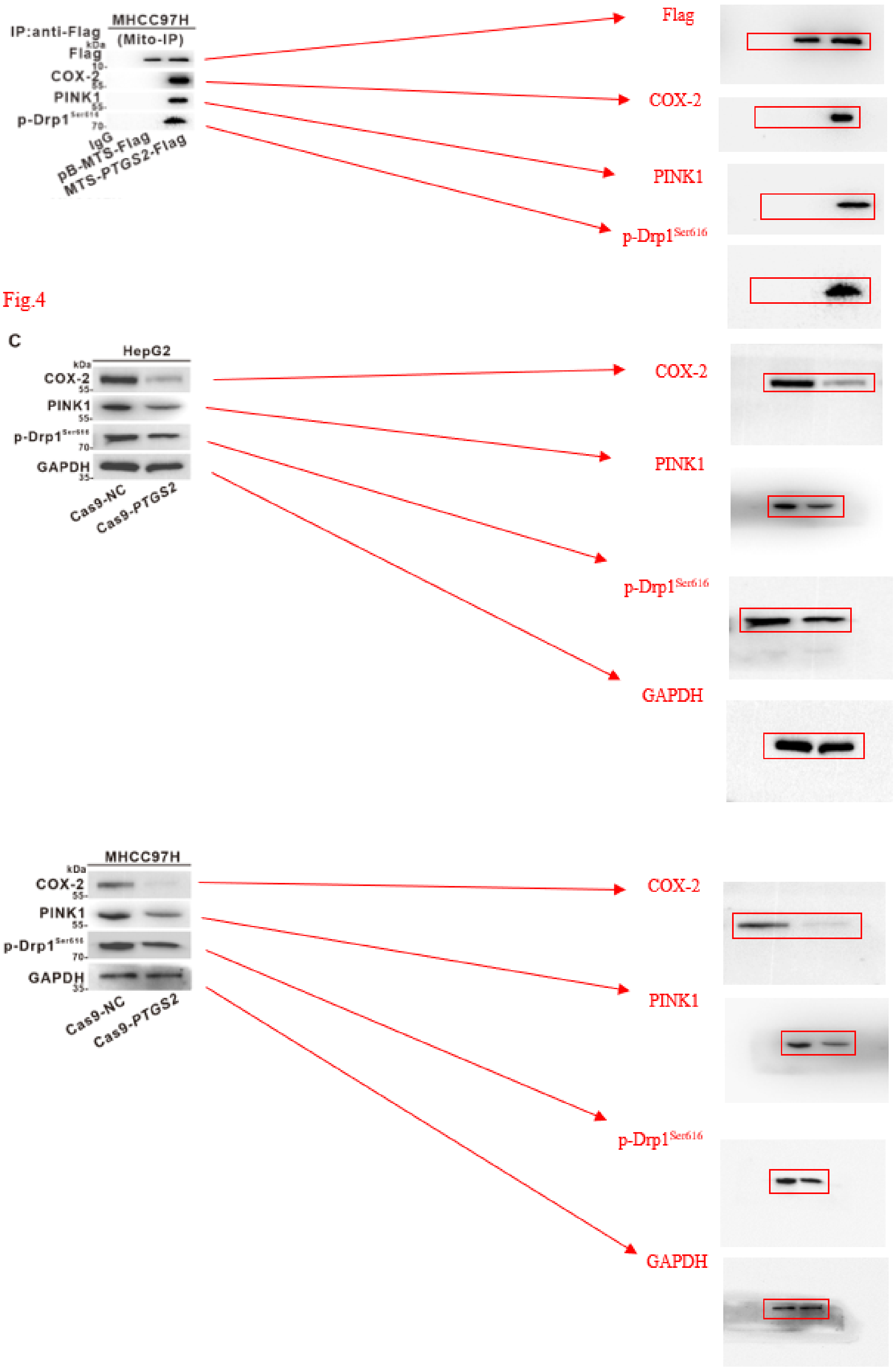
IP:anti-Flag
MHCC97H
(Mito-IP)
Flag
COX-2
PINK1
p-Drp1
IgG
pB-MTS-Flag
MTS-PTGS2-Flag
Flag
COX-2
PINK1
p-Drp1Ser616
C
HepG2
COX-2
PINK1
p-Drp1
GAPDH
Cas9-NC
Cas9-PTGS2
COX-2
PINK1
p-Drp1Ser616
GAPDH
MHCC97H
COX-2
PINK1
p-Drp1
GAPDH
Cas9-NC
Cas9-PTGS2
COX-2
PINK1
p-Drp1Ser616
GAPDH

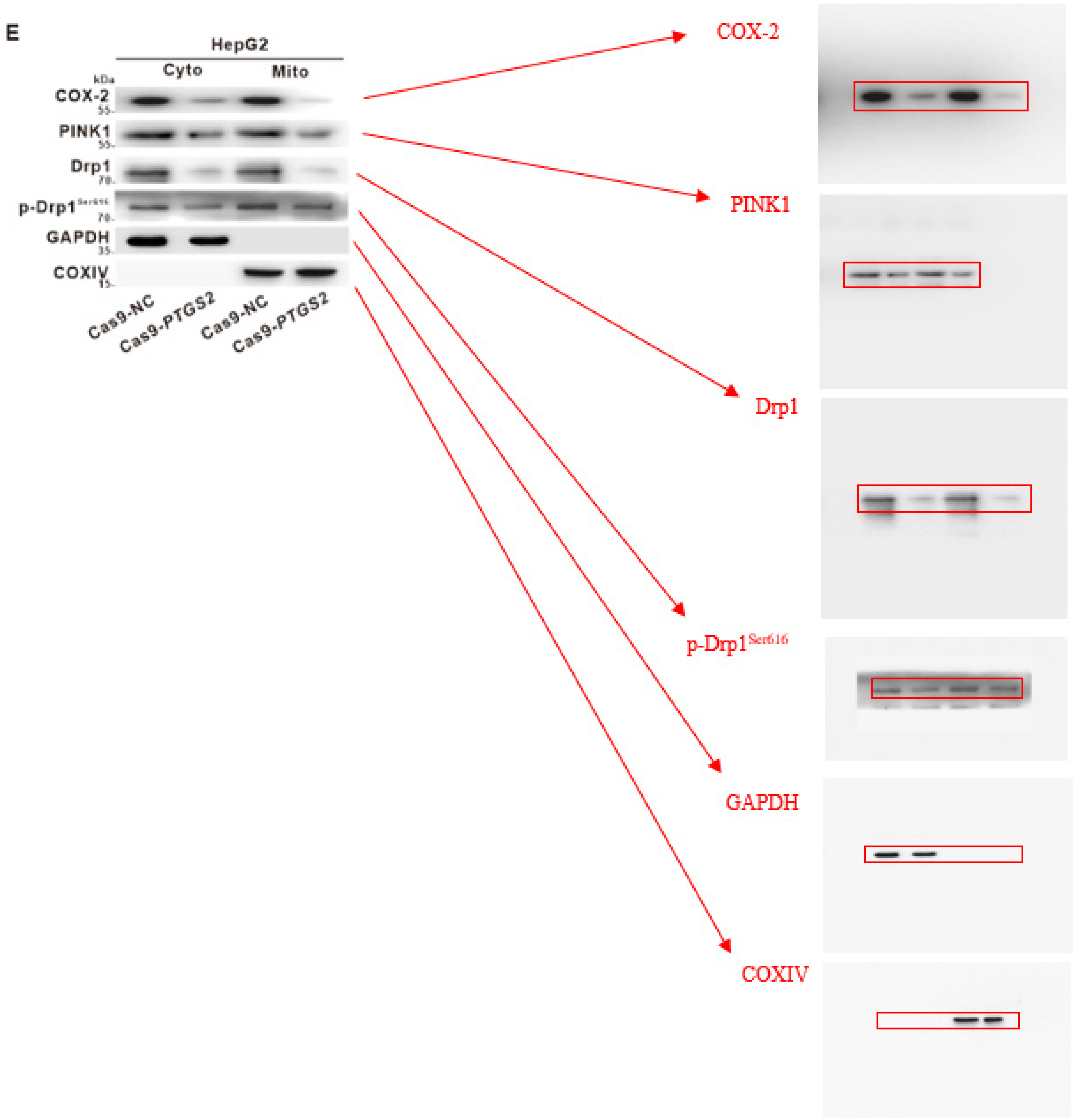
E
HepG2
Cyto
Mito
kDa
COX-2
55
PINK1
55
Drp1
70
p-Drp1 Ser616
70
GAPDH
35
COXIV
15
Cas9-NC
Cas9-PTGS2
Cas9-NC
Cas9-PTGS2
COX-2
PINK1
Drp1
p-Drp1 Ser616
GAPDH
COXIV

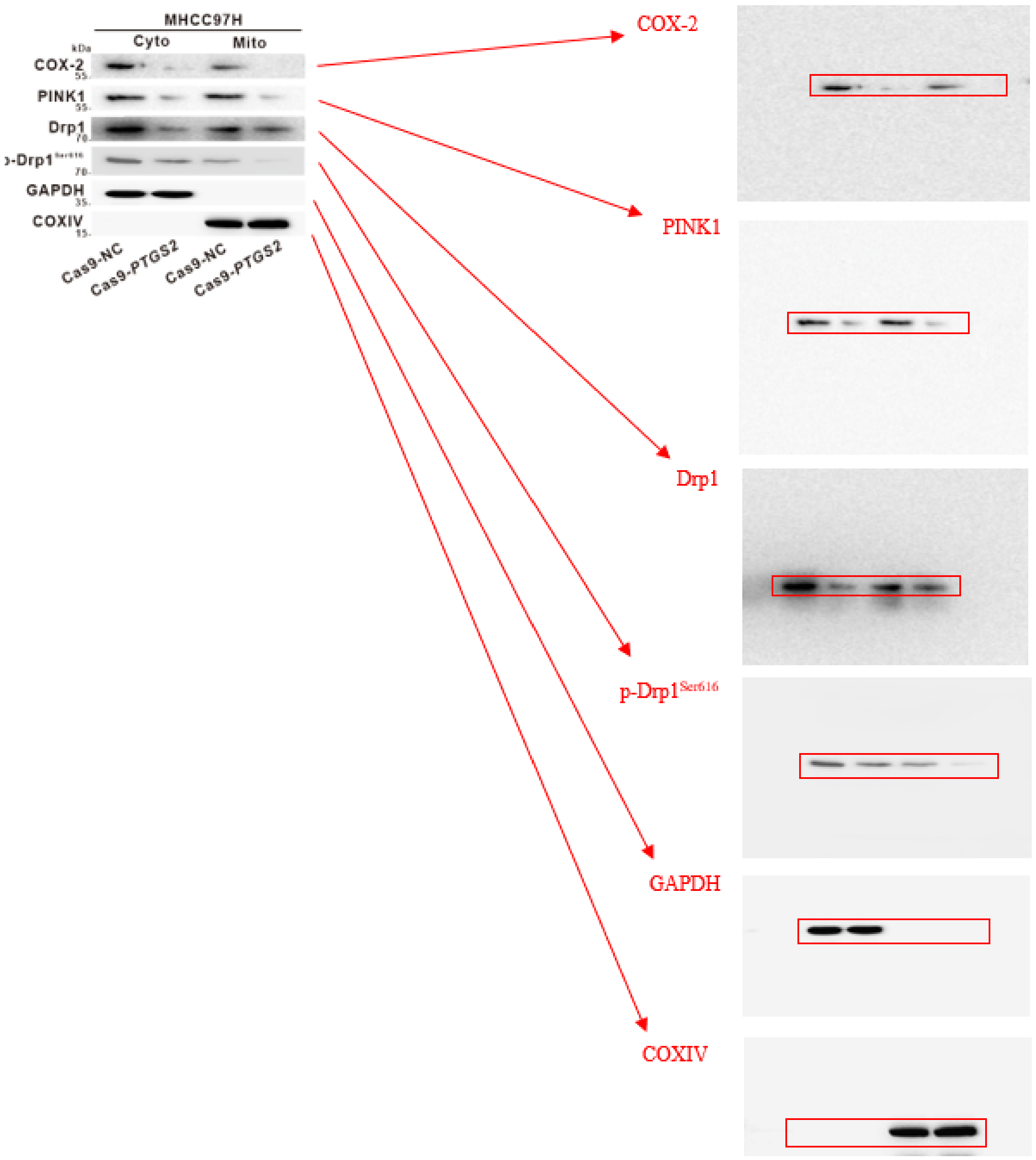
MHCC97H
Cyto
Mito
kDa
COX-2
55
PINK1
55
Drp1
70
p-Drp1 Ser616
70
GAPDH
35
COXIV
15
Cas9-NC
Cas9-PTGS2
Cas9-NC
Cas9-PTGS2
COX-2
PINK1
Drp1
p-Drp1 Ser616
GAPDH
COXIV

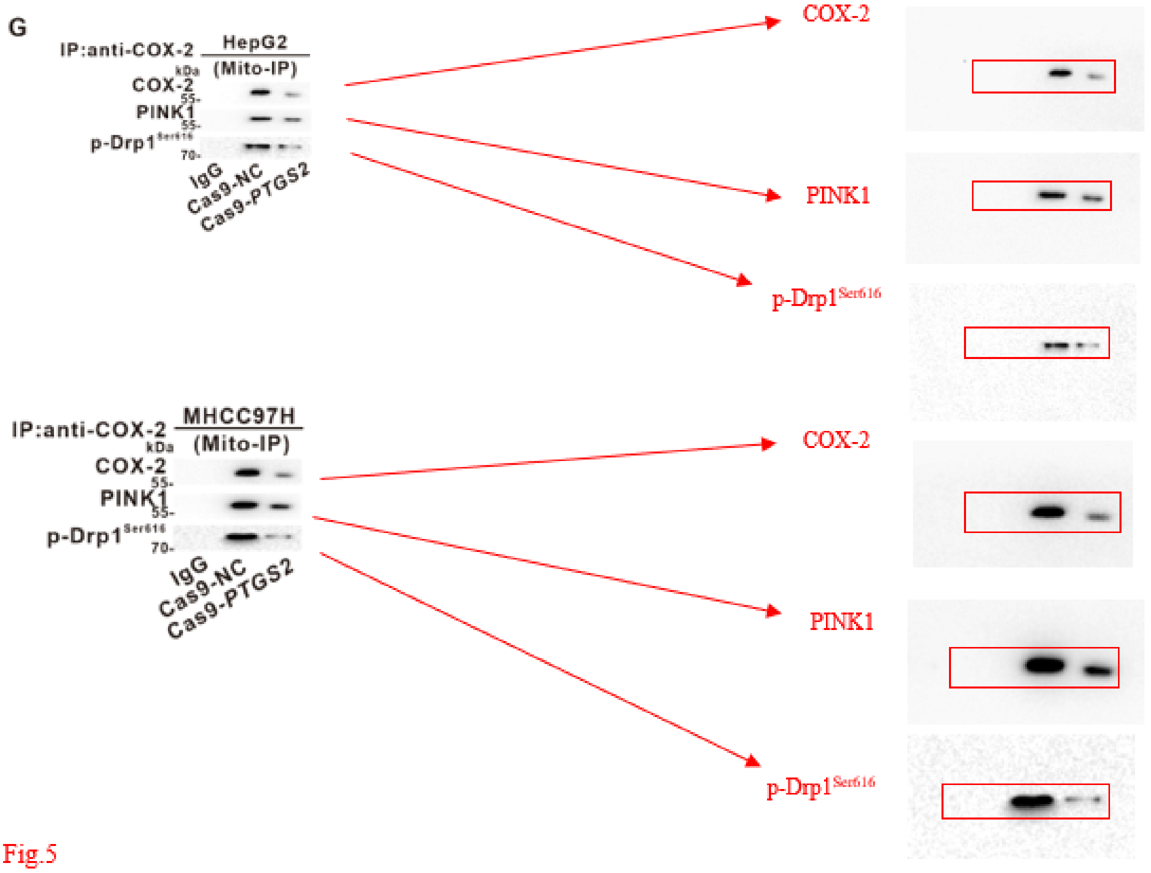
G
IP:anti-COX-2
HepG2
(Mito-IP)
kDa
COX-2
55-
PINK1
55-
p-Drp1Ser616
70-
IgG
Cas9-NC
Cas9-PTGS2
COX-2
PINK1
p-Drp1Ser616
IP:anti-COX-2
MHCC97H
(Mito-IP)
kDa
COX-2
55-
PINK1
55-
p-Drp1Ser616
70-
IgG
Cas9-NC
Cas9-PTGS2
COX-2
PINK1
p-Drp1Ser616

Fig.5

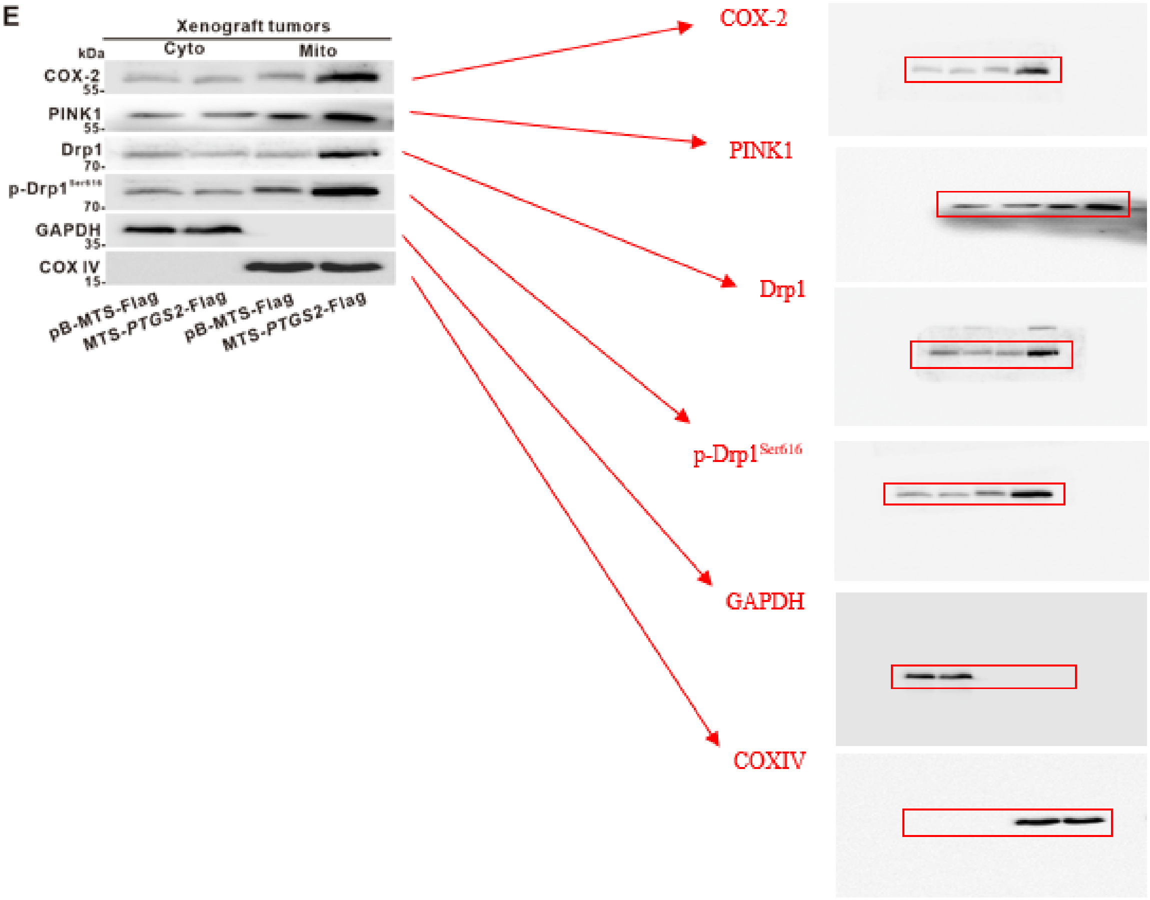
E
Xenograft tumors
kDa
Cyto
Mito
COX-2
55-
PINK1
55-
Drp1
70-
p-Drp1Ser616
70-
GAPDH
35-
COX IV
15-
pB-MTS-Flag
MTS-PTGS2-Flag
pB-MTS-Flag
MTS-PTGS2-Flag
COX-2
PINK1
Drp1
p-Drp1Ser616
GAPDH
COXIV

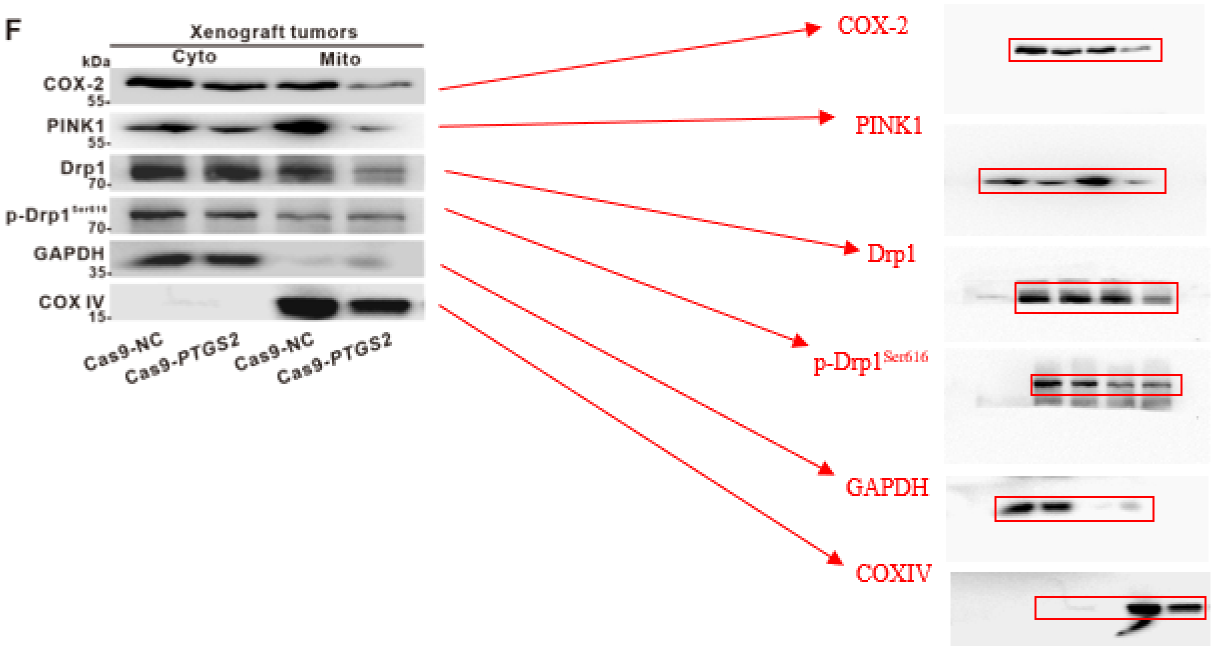
F
Xenograft tumors
Cyto
Mito
kDa
COX-2
55-
PINK1
55-
Drp1
70-
p-Drp1 Ser616
70-
GAPDH
35-
COX IV
15-
Cas9-NC
Cas9-PTGS2
Cas9-NC
Cas9-PTGS2
COX-2
PINK1
Drp1
p-Drp1 Ser616
GAPDH
COXIV

Fig.6

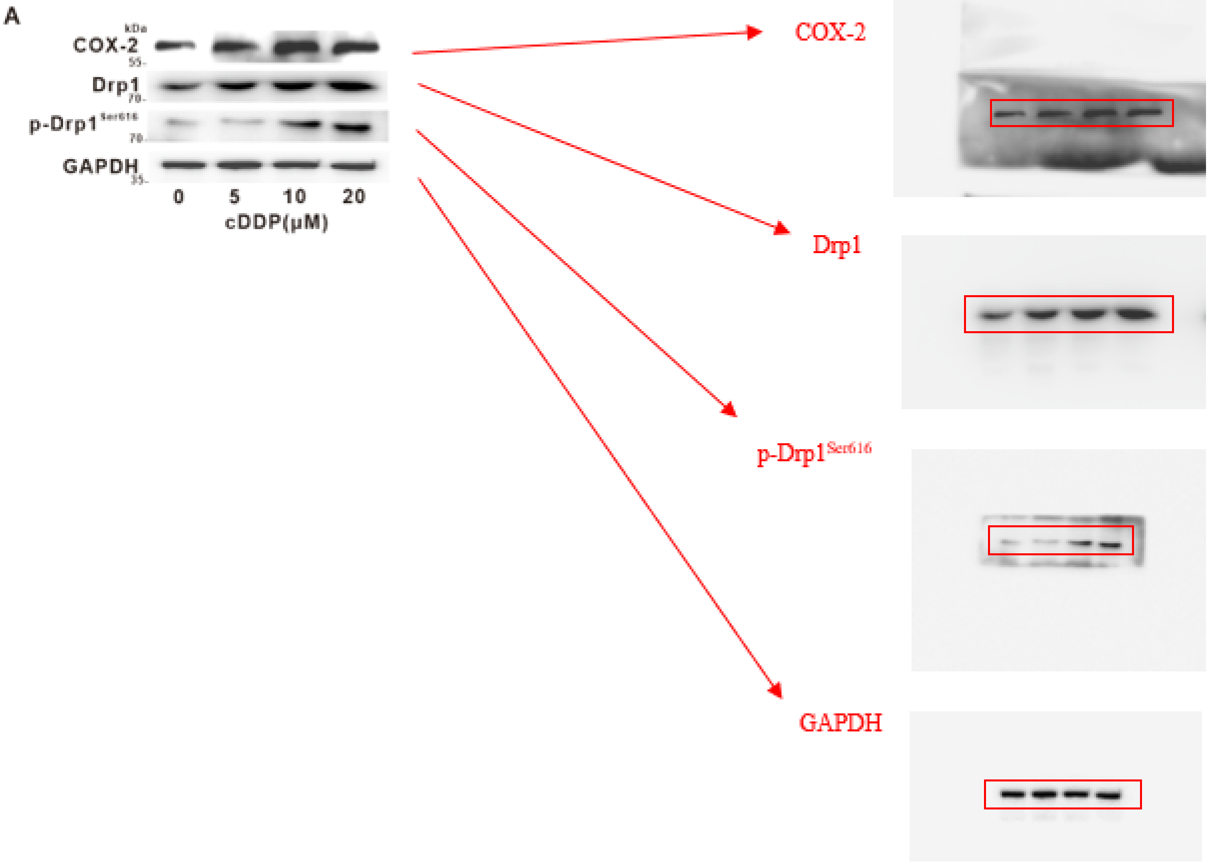
A
kDa
COX-2
55-
Drp1
70-
p-Drp1 Ser616
70
GAPDH
35-
0
5
10
20
cDDP(μM)
COX-2
Drp1
p-Drp1 Ser616
GAPDH

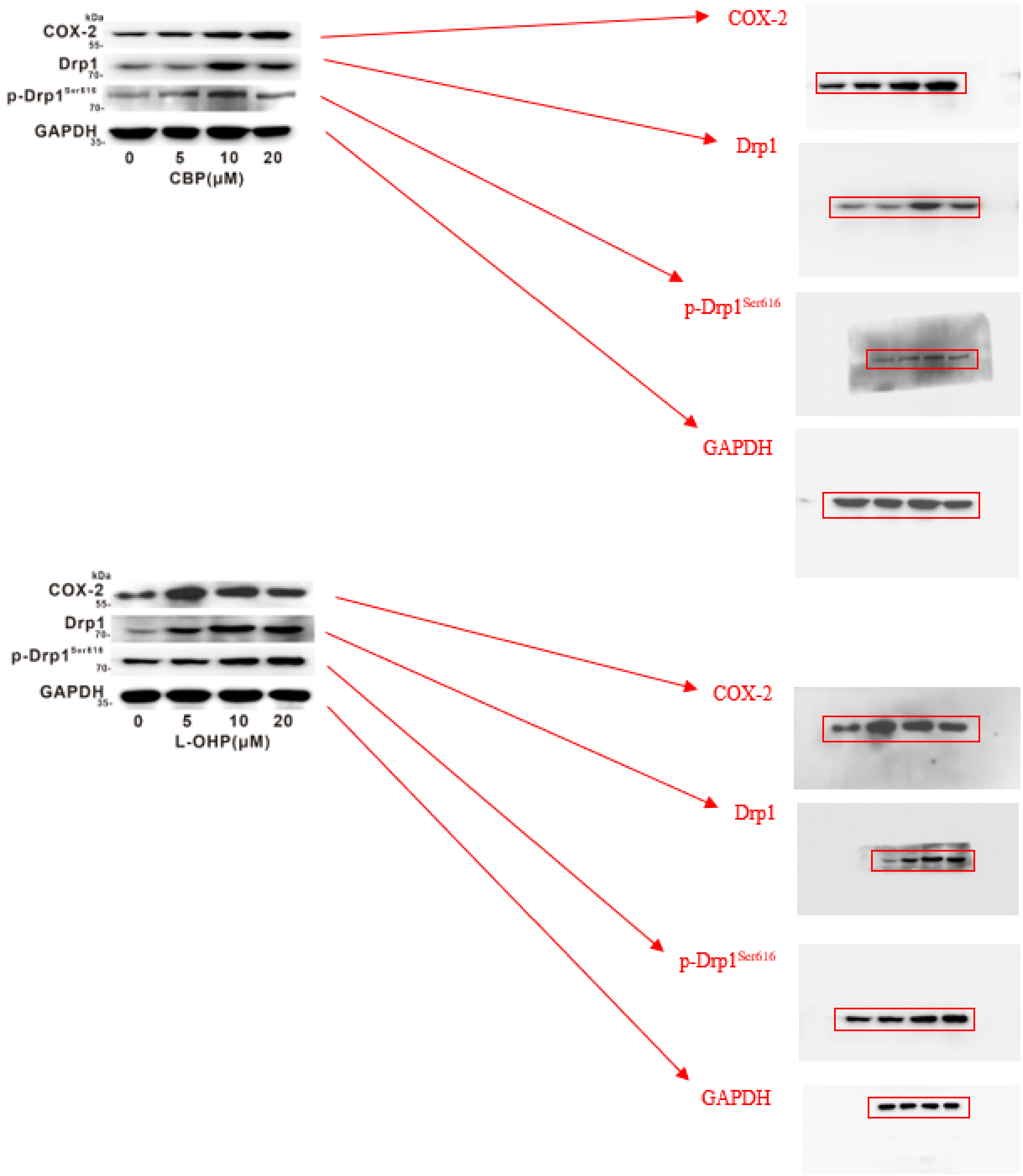
COX-2
Drp1
p-Drp1Ser616
GAPDH
0 5 10 20
CBP(μM)
COX-2
Drp1
p-Drp1Ser616
GAPDH
COX-2
Drp1
p-Drp1Ser616
GAPDH
0 5 10 20
L-OHP(μM)
COX-2
Drp1
p-Drp1Ser616
GAPDH

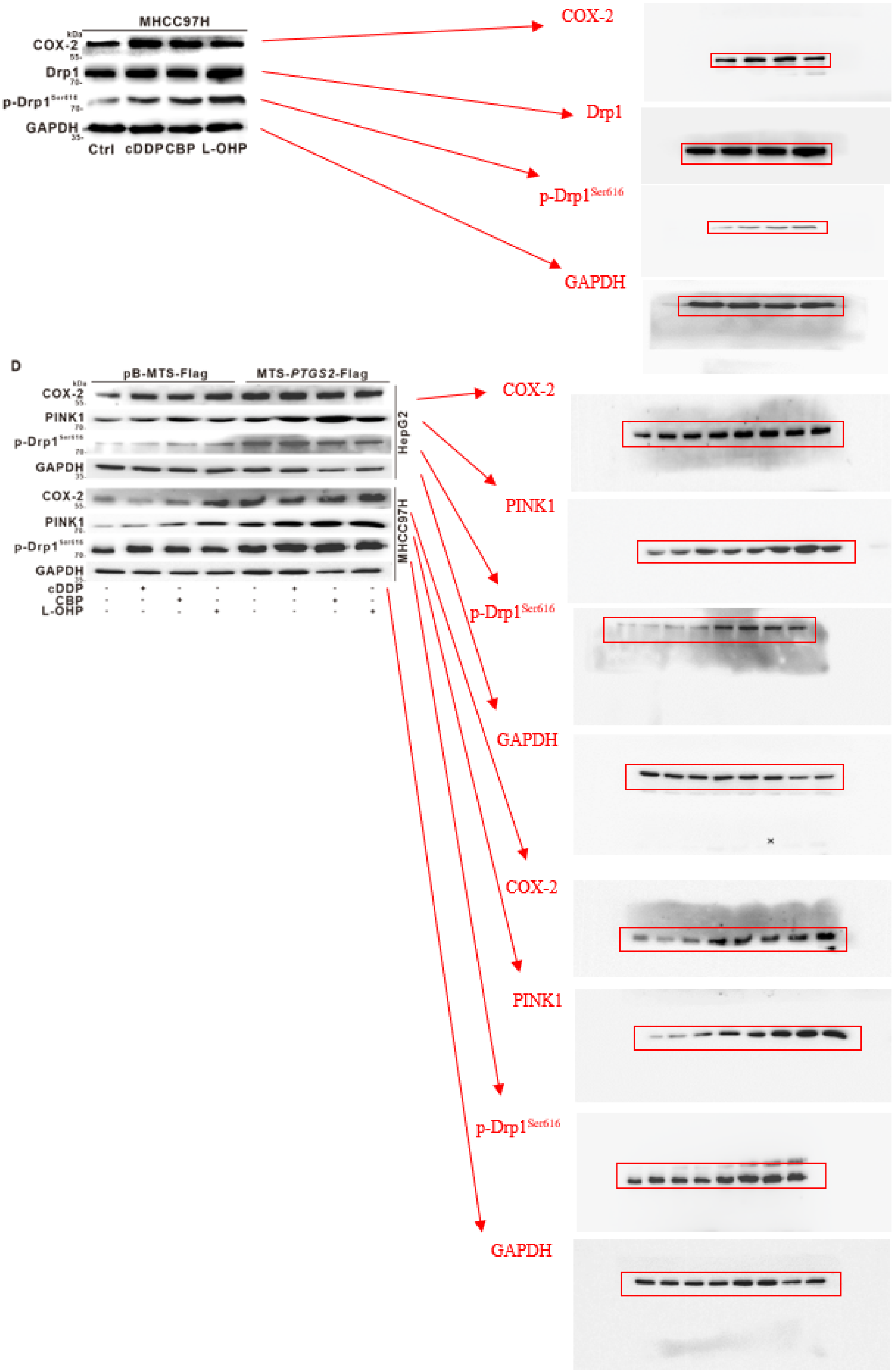
MHCC97H
COX-2
Drp1
p-Drp1Ser616
GAPDH
Ctrl
cDDP
CBP
L-OHP
D
pB-MTS-Flag
MTS-PTGS2-Flag
PINK1
HepG2
MHCC97H
cDDP
CBP
L-OHP
COX-2
Drp1
p-Drp1Ser616
GAPDH
COX-2
PINK1
p-Drp1Ser616
GAPDH
COX-2
PINK1
p-Drp1Ser616
GAPDH

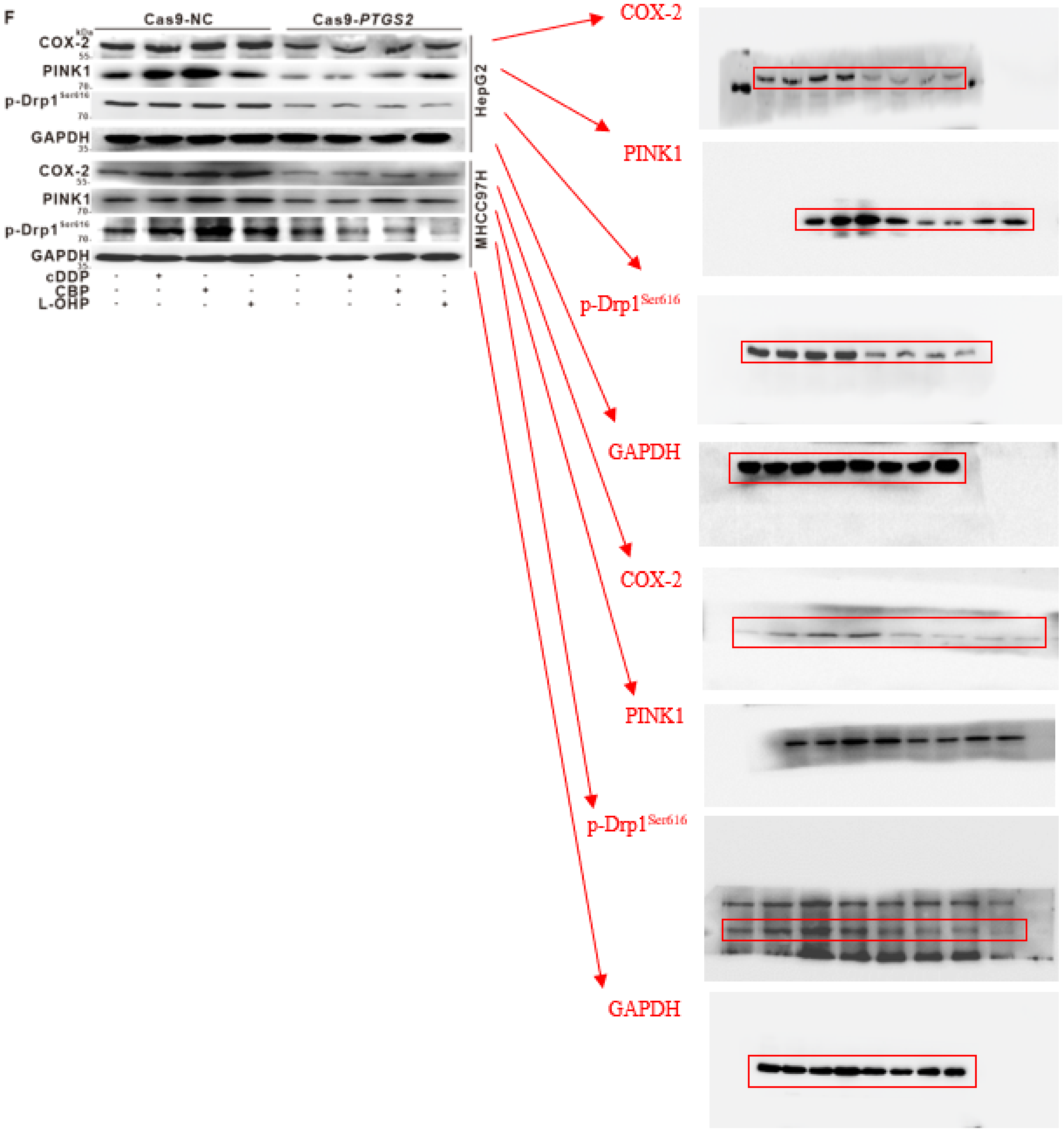
F
Cas9-NC
Cas9-PTGS2
COX-2
PINK1
p-Drp1 Ser616
GAPDH
HepG2
COX-2
PINK1
p-Drp1 Ser616
GAPDH
MHCC97H
cDDP
CBP
L-OHP
COX-2
PINK1
p-Drp1 Ser616
GAPDH
COX-2
PINK1
p-Drp1 Ser616
GAPDH

Fig. 7

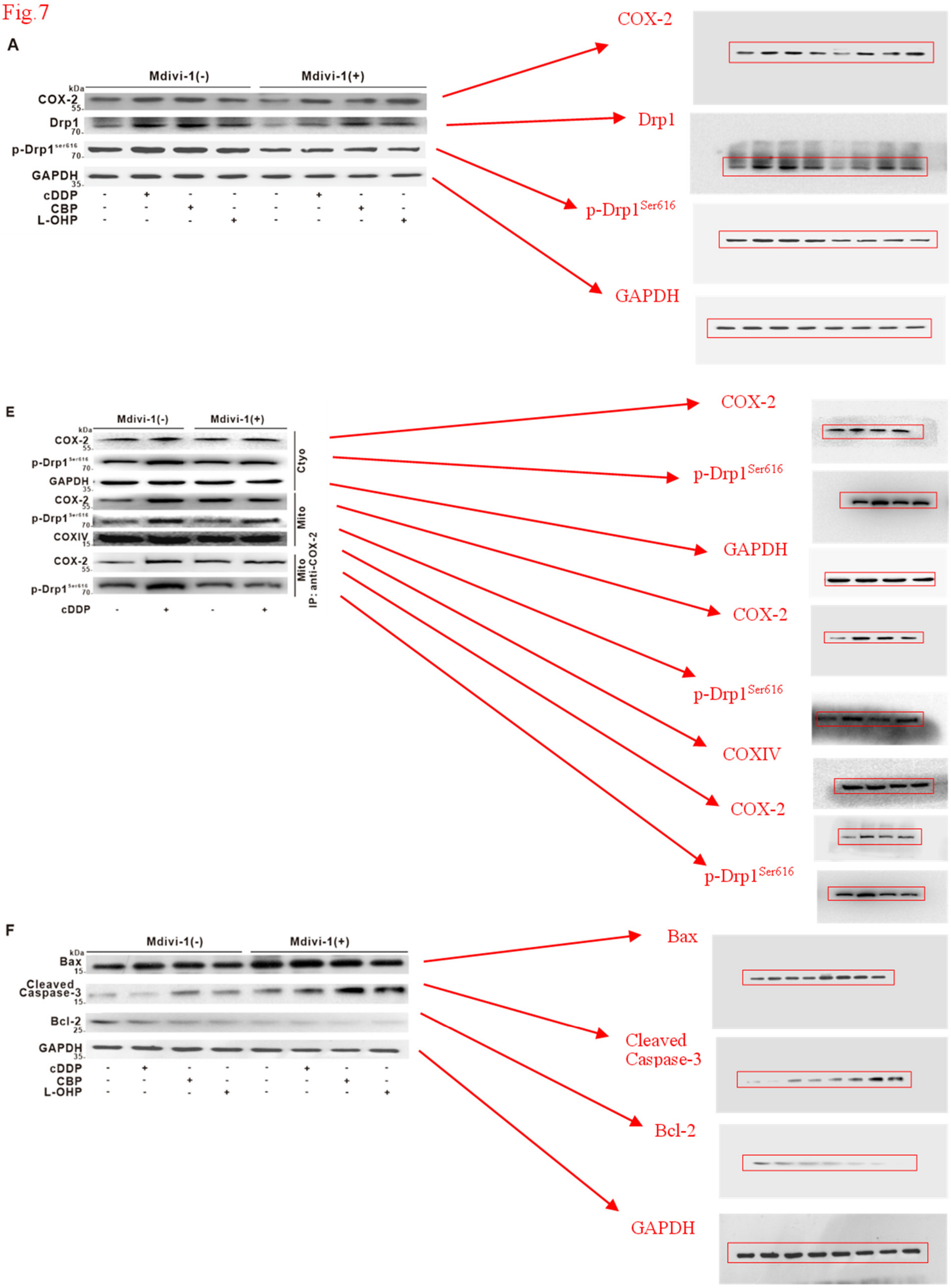
A
Mdivi-1(-)
Mdivi-1(+)
kDa
COX-2
Drp1
p-Drp1ser616
GAPDH
cDDP
CBP
L-OHP
COX-2
Drp1
p-Drp1Ser616
GAPDH
E
Mdivi-1(-)
Mdivi-1(+)
COX-2
p-Drp1ser616
GAPDH
COXIV
Ctyo
Mito
Mito IP: anti-COX-2
cDDP
COX-2
p-Drp1Ser616
GAPDH
COXIV
F
Mdivi-1(-)
Mdivi-1(+)
Bax
Cleaved Caspase-3
Bcl-2
GAPDH
cDDP
CBP
L-OHP
Bax
Cleaved Caspase-3
Bcl-2
GAPDH

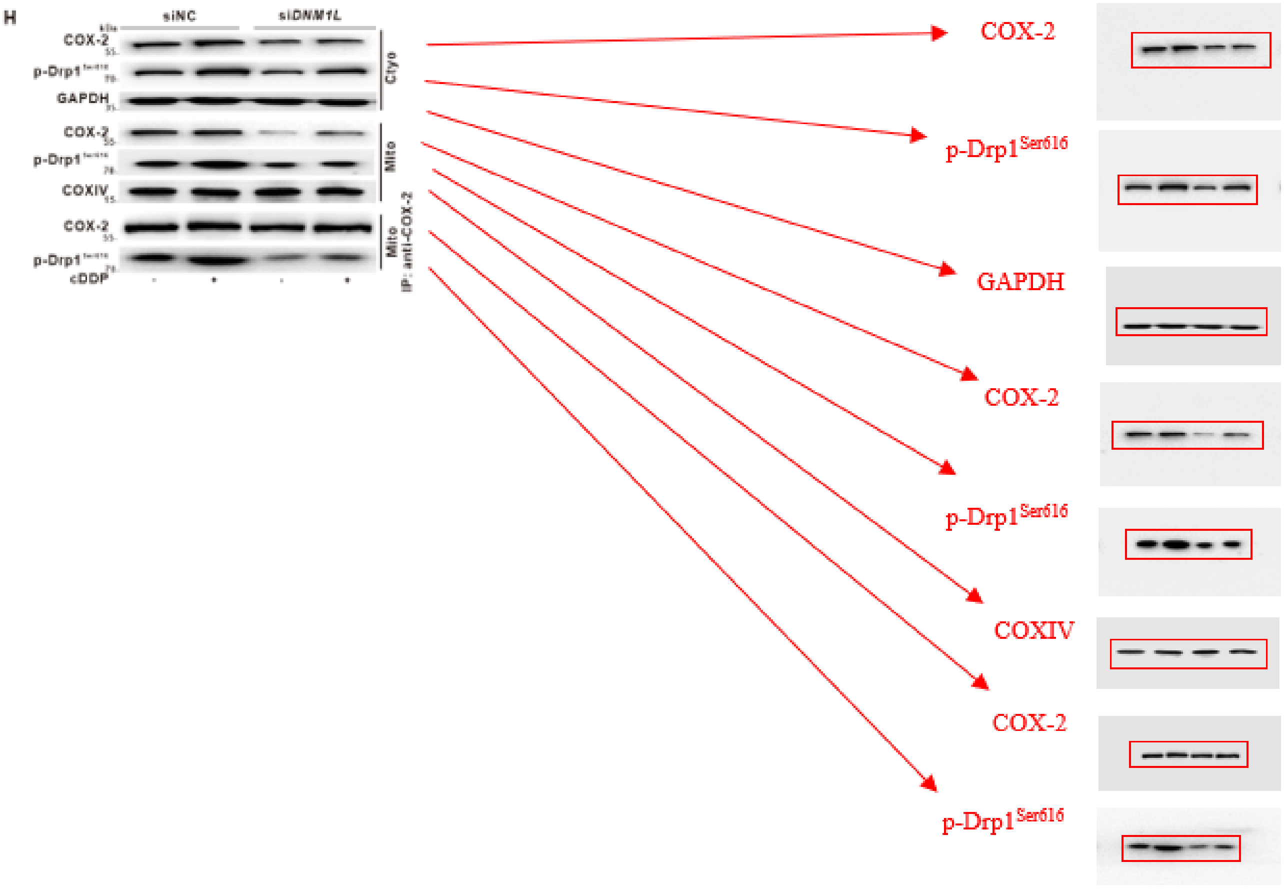
H
siNC
siDNM1L
COX-2
p-Drp1
GAPDH
COX-2
p-Drp1
COXIV
COX-2
p-Drp1
cDDP
Ctyo
Mito
Mito
IP: anti-COX-2
COX-2
p-Drp1 Ser616
GAPDH
COX-2
p-Drp1 Ser616
COXIV
COX-2
p-Drp1 Ser616

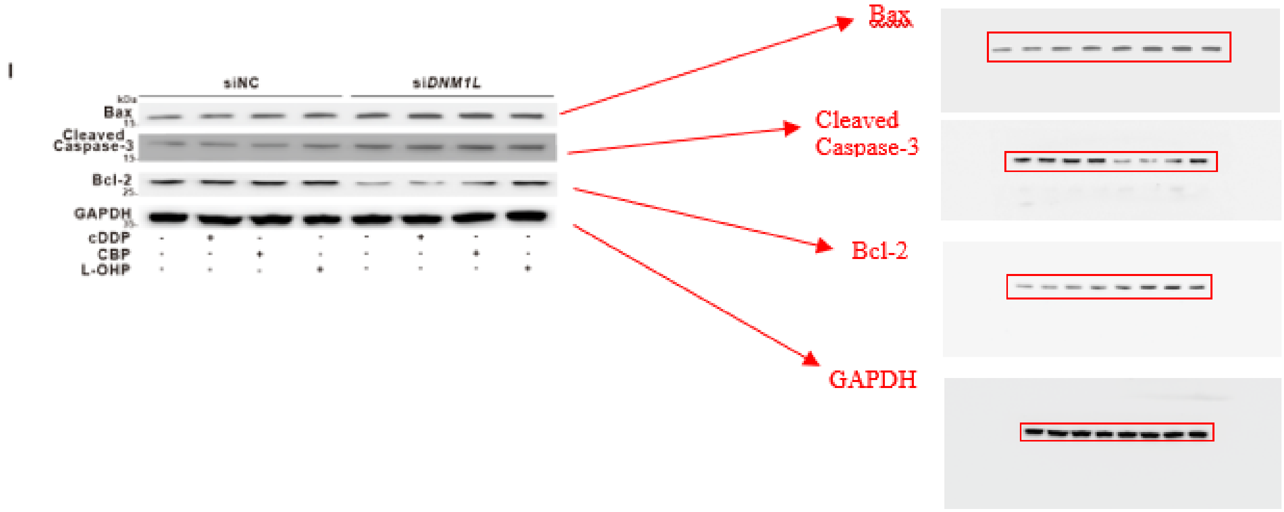
I
siNC
siDNM1L
Bax
Cleaved Caspase-3
Bcl-2
GAPDH
cDDP
CBP
L-OHP
Bax
Cleaved Caspase-3
Bcl-2
GAPDH

Fig.8

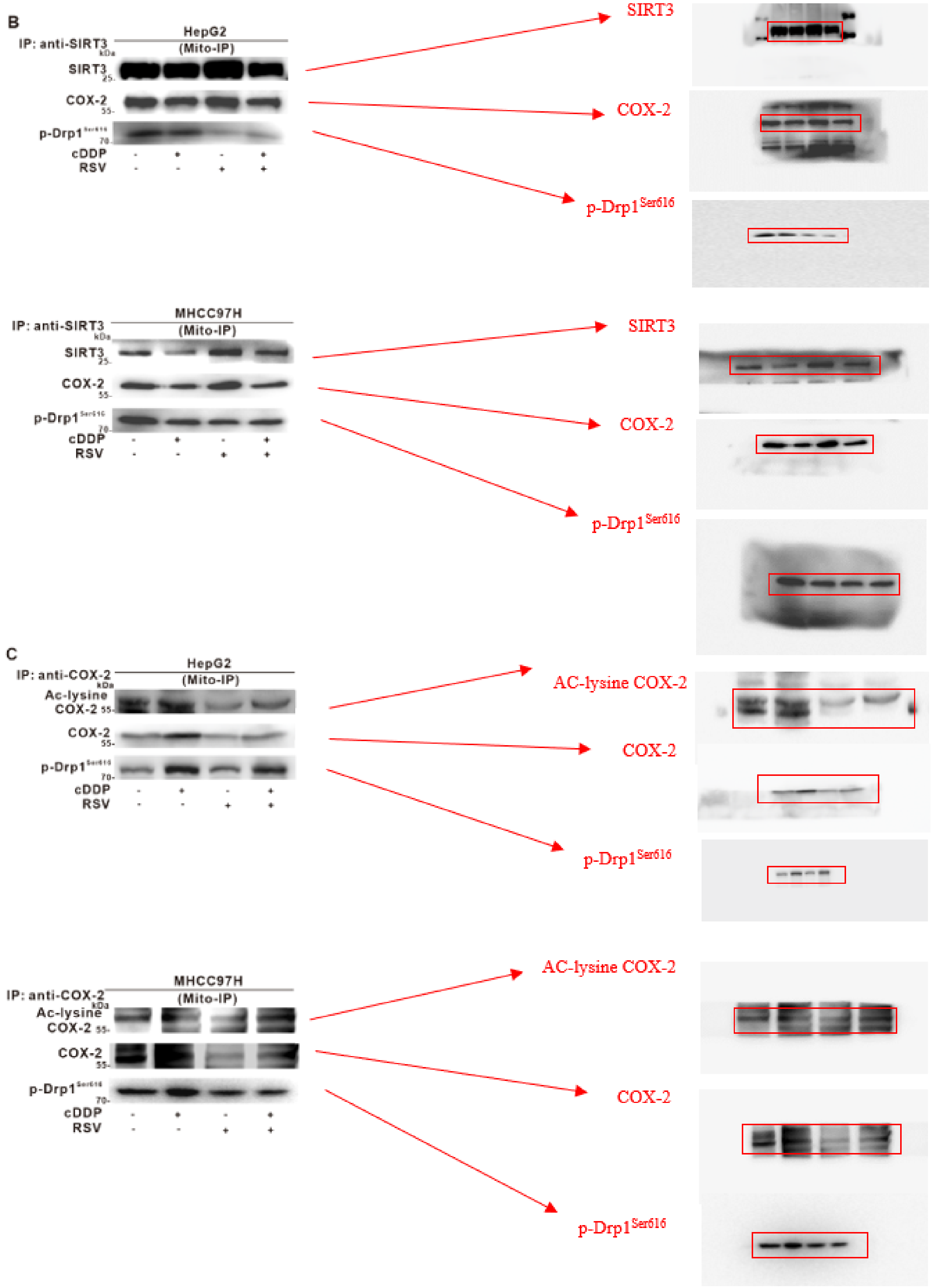
B
IP: anti-SIRT3
HepG2
(Mito-IP)
SIRT3
COX-2
p-Drp1
cDDP
RSV
SIRT3
COX-2
p-Drp1 Ser616
IP: anti-SIRT3
MHCC97H
(Mito-IP)
SIRT3
COX-2
p-Drp1
cDDP
RSV
SIRT3
COX-2
p-Drp1 Ser616
C
IP: anti-COX-2
HepG2
(Mito-IP)
Ac-lysine
COX-2
COX-2
p-Drp1
cDDP
RSV
AC-lysine COX-2
COX-2
p-Drp1 Ser616
IP: anti-COX-2
MHCC97H
(Mito-IP)
Ac-lysine
COX-2
COX-2
p-Drp1
cDDP
RSV
AC-lysine COX-2
COX-2
p-Drp1 Ser616

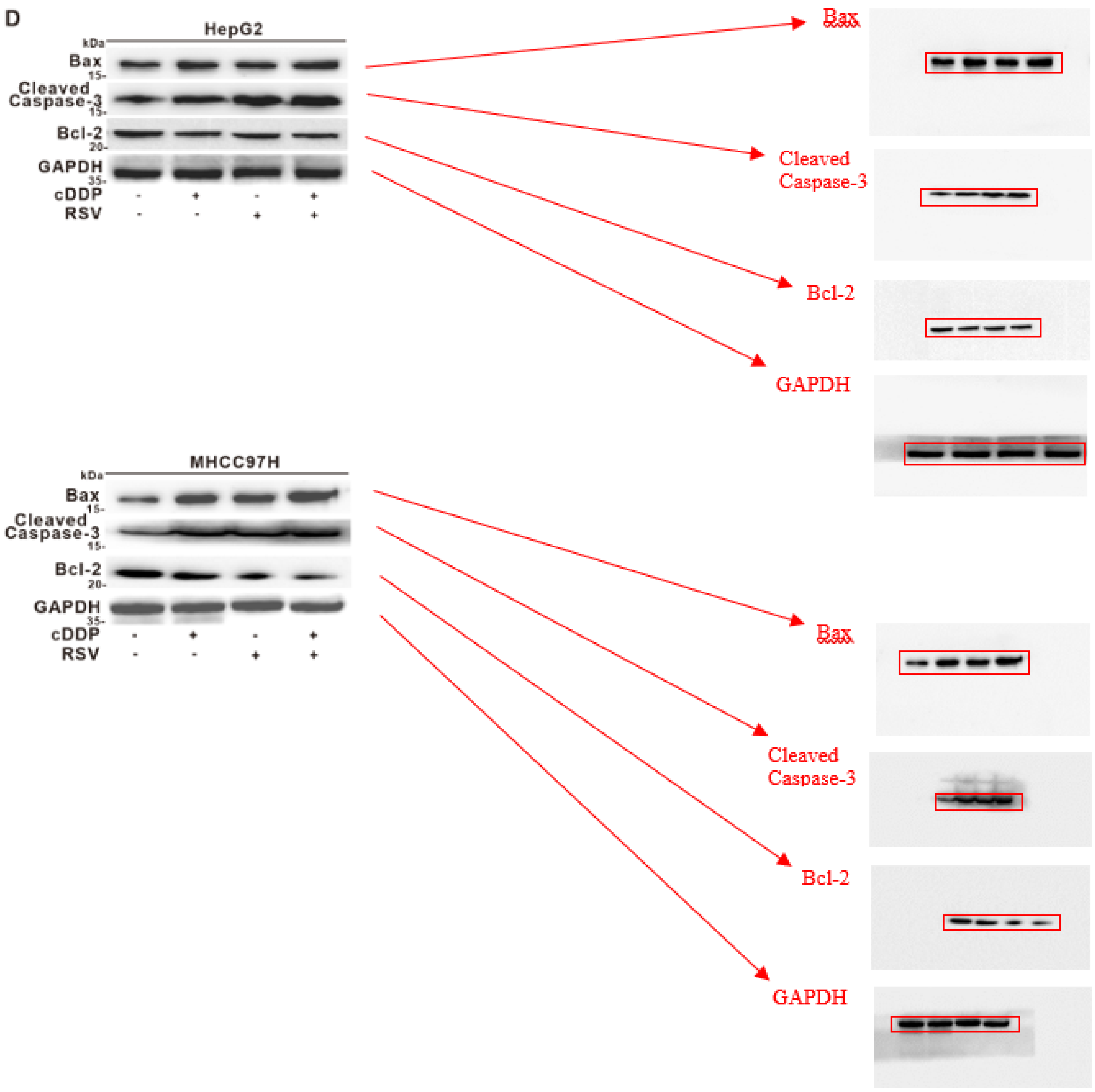
D
HepG2
kDa
Bax
15-
Cleaved
Caspase-3
15-
Bcl-2
20-
GAPDH
35-
cDDP - + - +
RSV - - + +
MHCC97H
kDa
Bax
15-
Cleaved
Caspase-3
15-
Bcl-2
20-
GAPDH
35-
cDDP - + - +
RSV - - + +
Bax
Cleaved
Caspase-3
Bcl-2
GAPDH
Bax
Cleaved
Caspase-3
Bcl-2
GAPDH

F

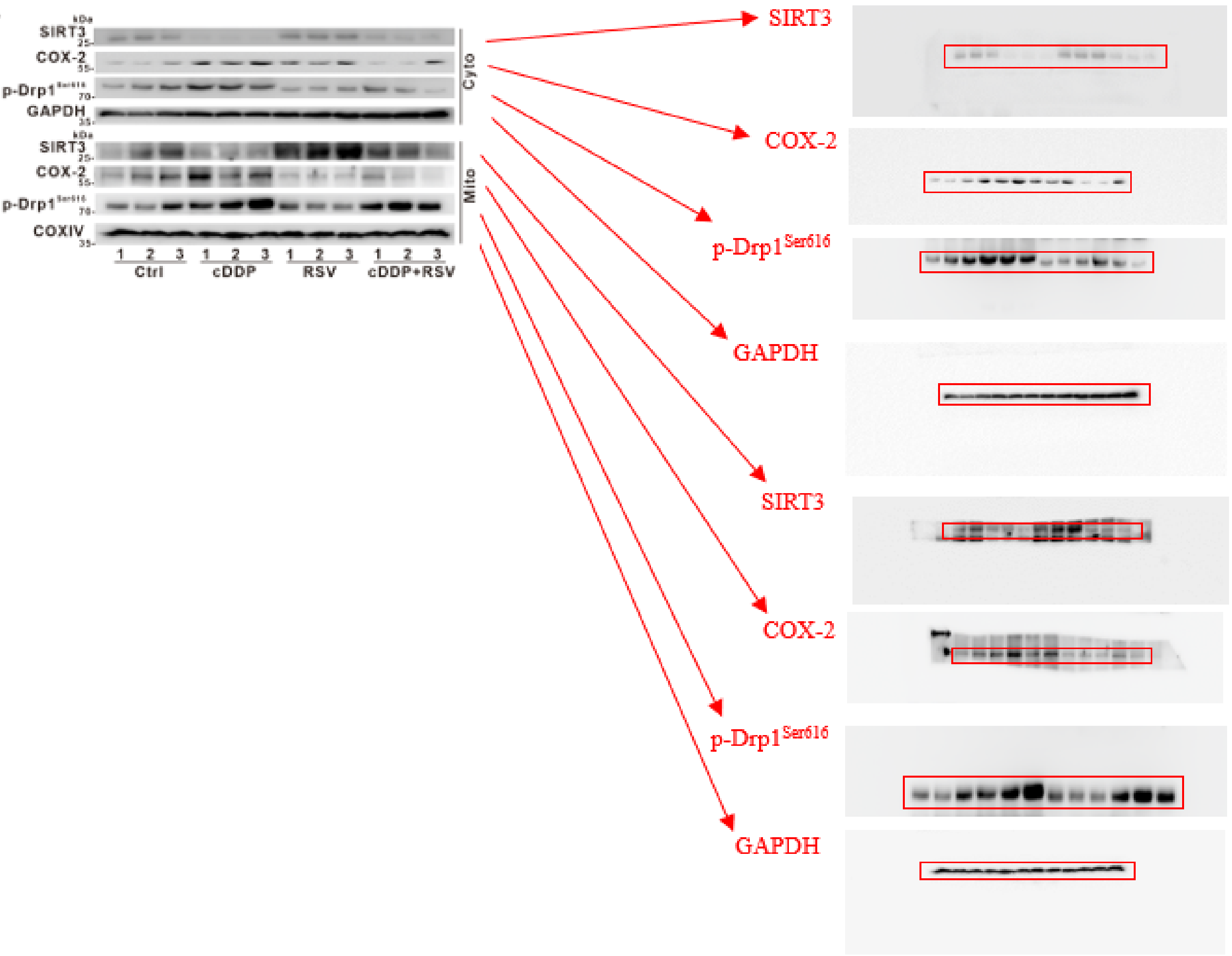
kDa
SIRT3
25-
COX-2
55-
p-Drp1Ser616
70-
GAPDH
35-
Cyto
SIRT3
25-
COX-2
55-
p-Drp1Ser616
70-
COXIV
35-
Mito
1 2 3 1 2 3 1 2 3 1 2 3
Ctrl
cDDP
RSV
cDDP+RSV
SIRT3
COX-2
p-Drp1Ser616
GAPDH
SIRT3
COX-2
p-Drp1Ser616
GAPDH

H

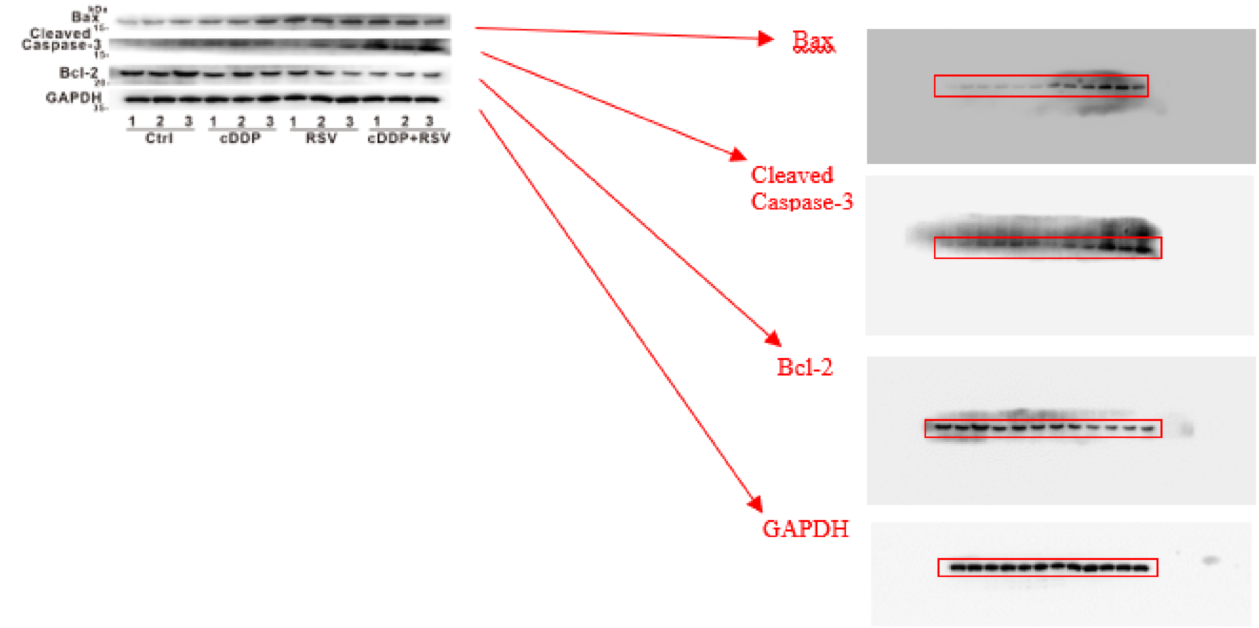
kDa
Bax
15-
Cleaved Caspase-3
15-
Bcl-2
20-
GAPDH
35-
1 2 3 1 2 3 1 2 3 1 2 3
Ctrl
cDDP
RSV
cDDP+RSV
Bax
Cleaved Caspase-3
Bcl-2
GAPDH

Fig.S3

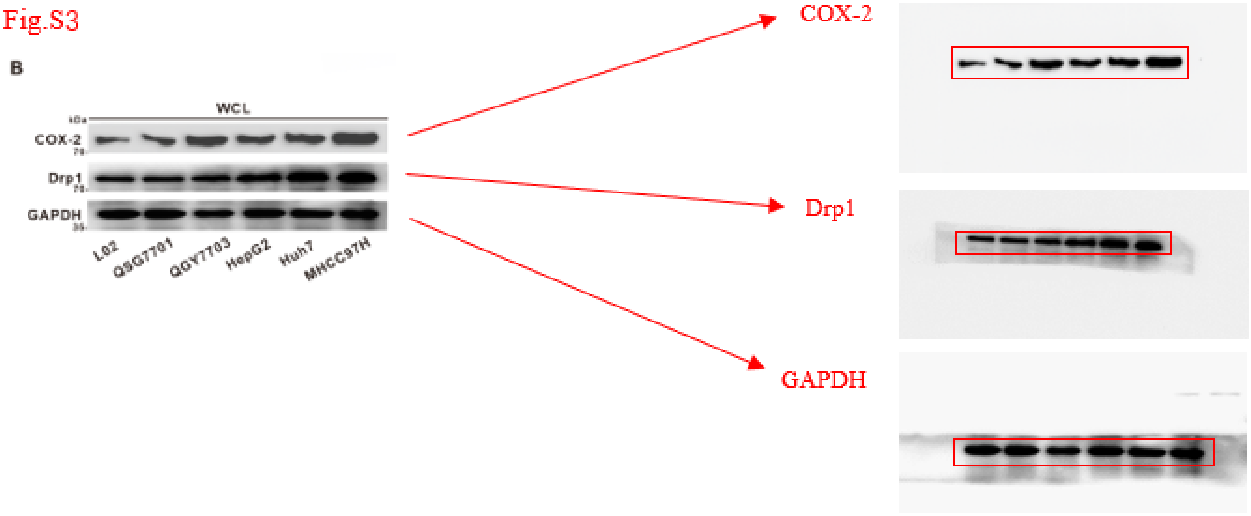

Fig.S4

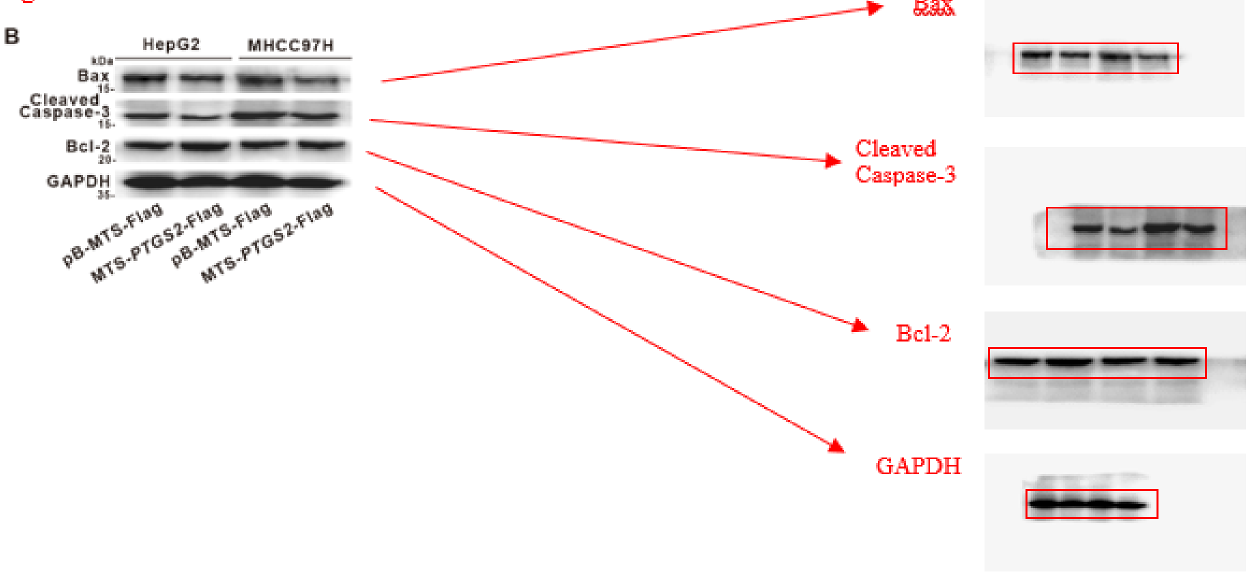

Fig.S5

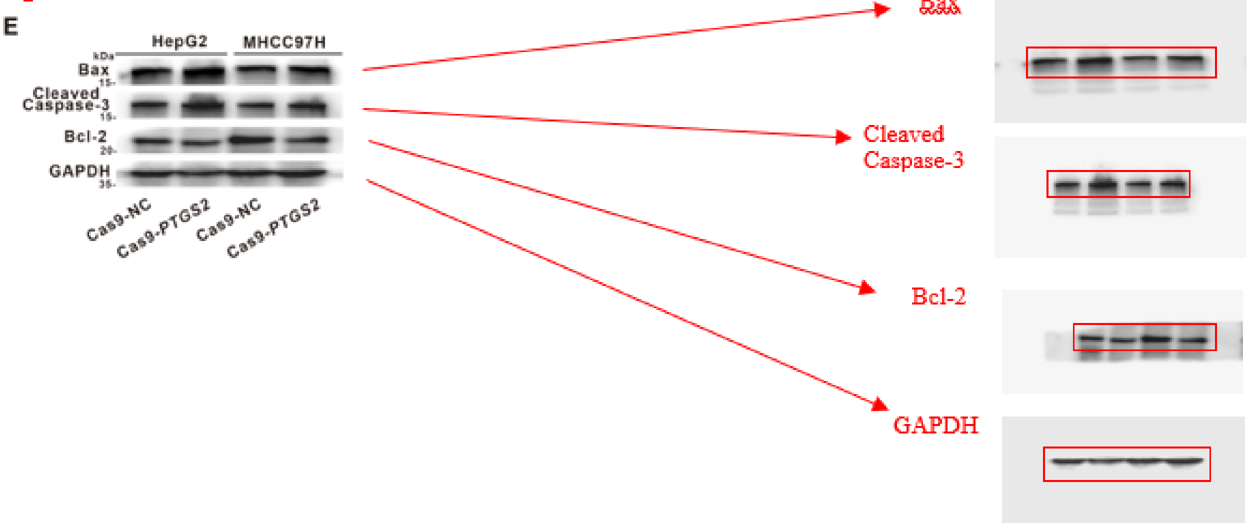

Fig.S7

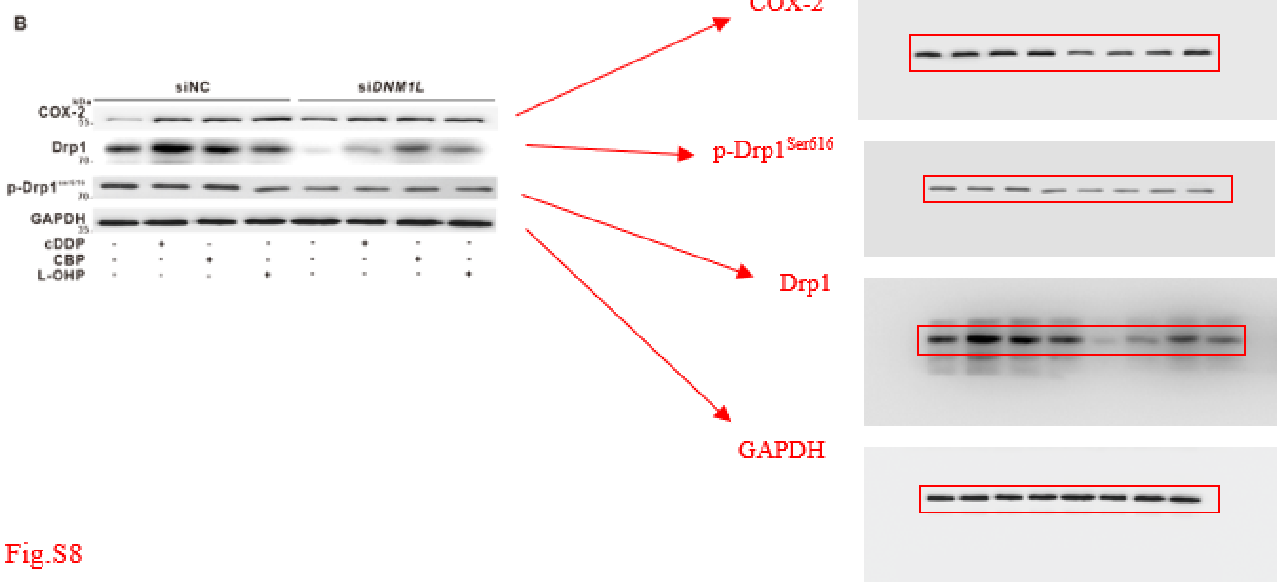
B
siNC
siDNM1L
COX-2
Drp1
p-Drp1
GAPDH
cDDP
CBP
L-OHP
COX-2
p-Drp1 Ser616
Drp1
GAPDH

Fig.S8

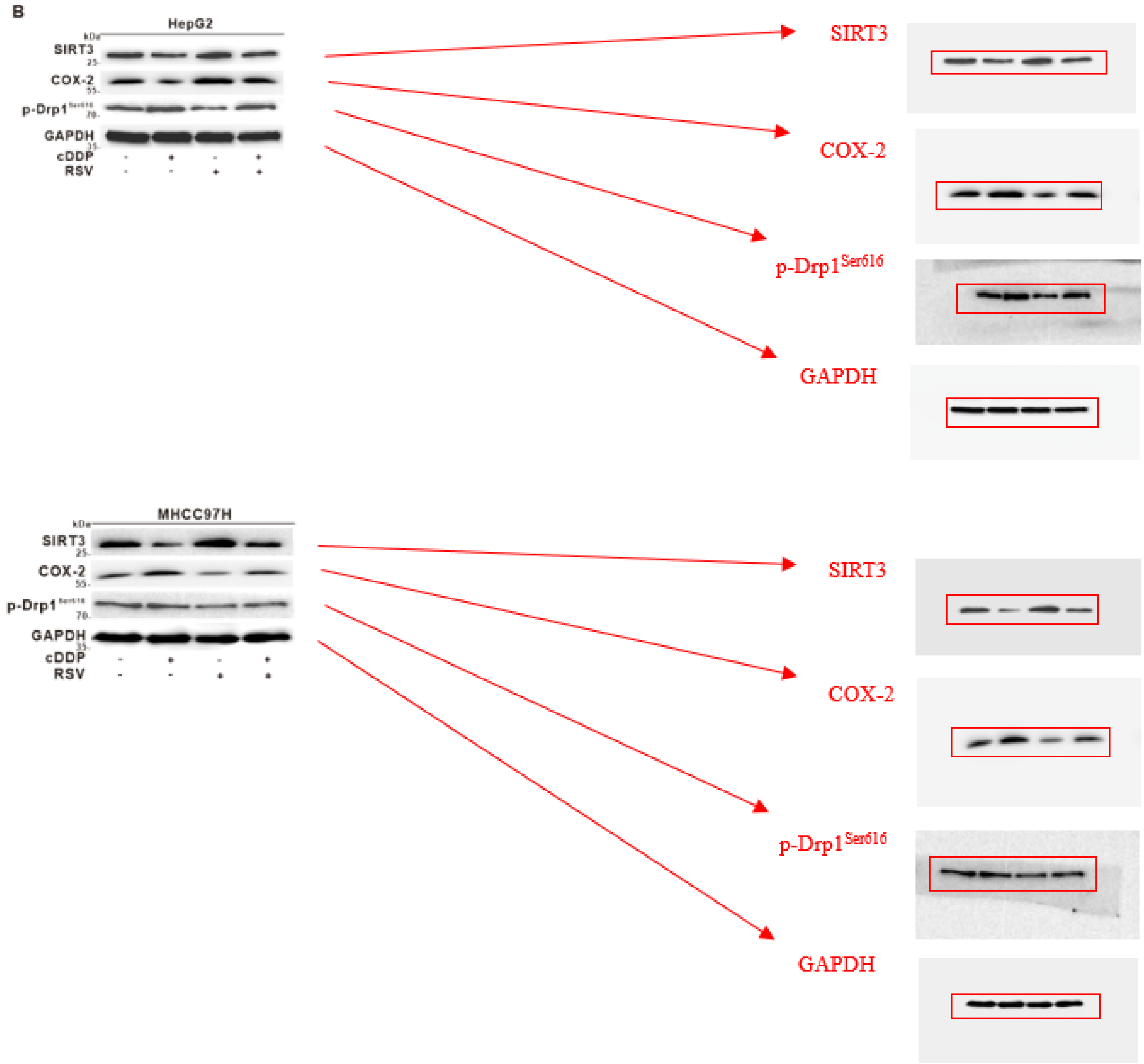
B
HepG2
SIRT3
COX-2
p-Drp1
GAPDH
cDDP
RSV
SIRT3
COX-2
p-Drp1 Ser616
GAPDH
MHCC97H
SIRT3
COX-2
p-Drp1
GAPDH
cDDP
RSV
SIRT3
COX-2
p-Drp1 Ser616
GAPDH

F

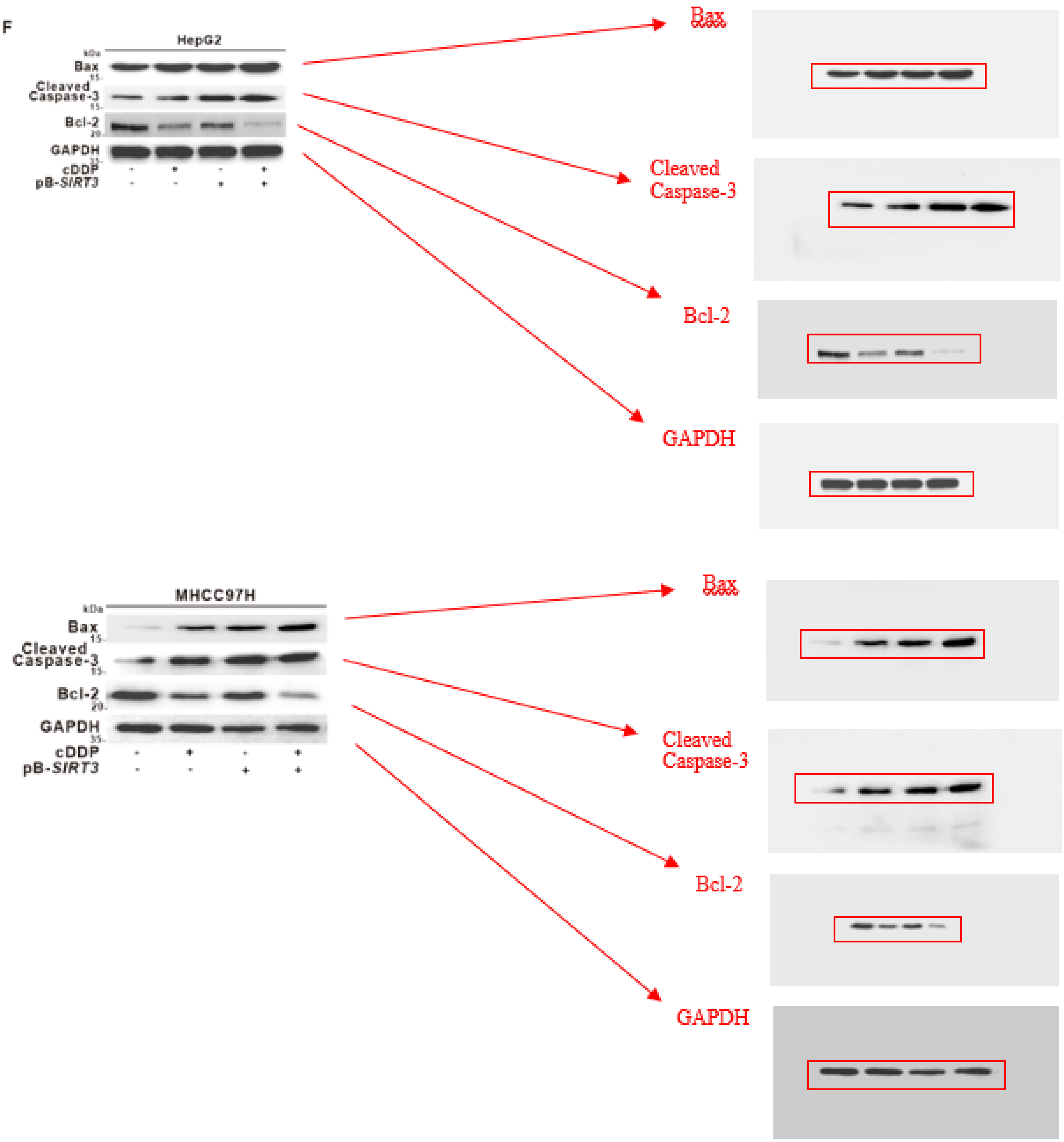
HepG2
kDa
Bax
Cleaved Caspase-3
Bcl-2
GAPDH
cDDP
pB-SIRT3
Bax
Cleaved Caspase-3
Bcl-2
GAPDH
MHCC97H
kDa
Bax
Cleaved Caspase-3
Bcl-2
GAPDH
cDDP
pB-SIRT3
Bax
Cleaved Caspase-3
Bcl-2
GAPDH

I

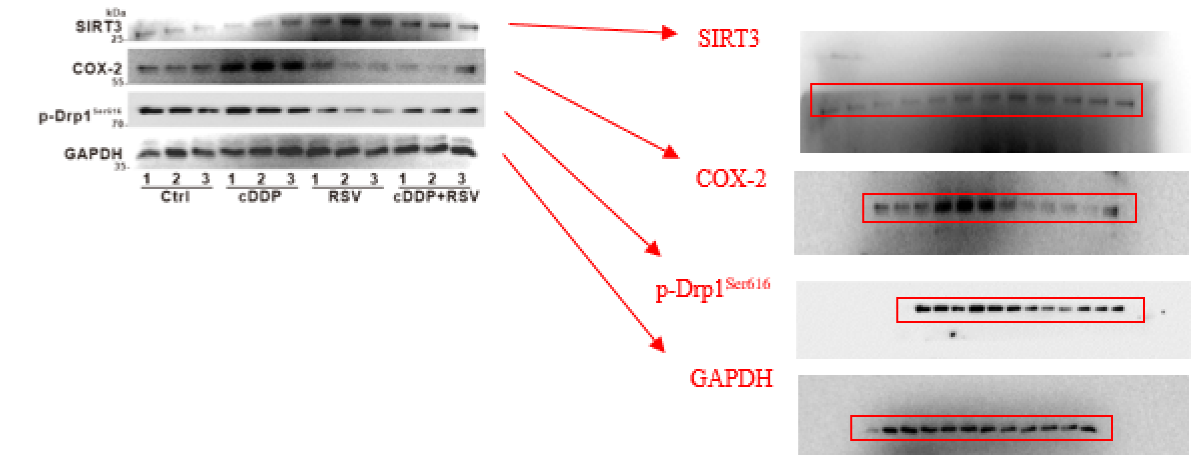
kDa
SIRT3
COX-2
p-Drp1 Ser616
GAPDH
1 2 3 1 2 3 1 2 3 1 2 3
Ctrl
cDDP
RSV
cDDP+RSV
SIRT3
COX-2
p-Drp1 Ser616
GAPDH

Figure S9. The original full scan of each WB (as shown below, right panels) and the cropped area had been indicated (in red rectangle). Western blot bands were visualized using the Azure Biosystems. The whole membranes were shown in supplementary materials.

The corresponding protein quantification bar graphs for Fig.1D:

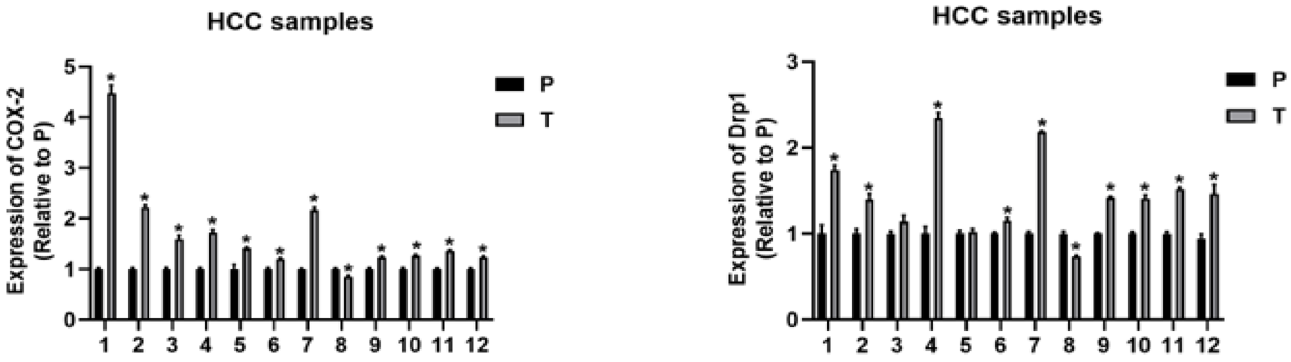

The corresponding protein quantification bar graphs for Fig.2C:

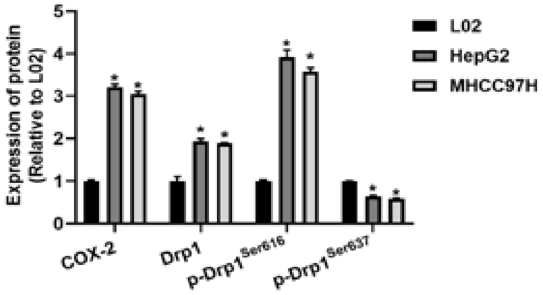

The corresponding protein quantification bar graphs for Fig.2D:

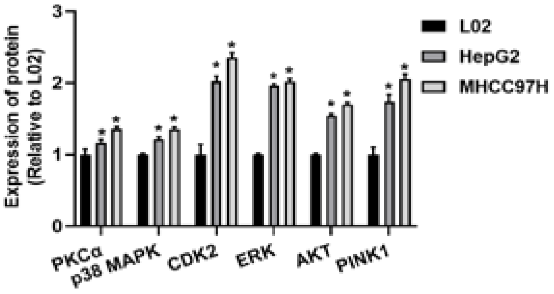

The corresponding protein quantification bar graphs for Fig.2E:

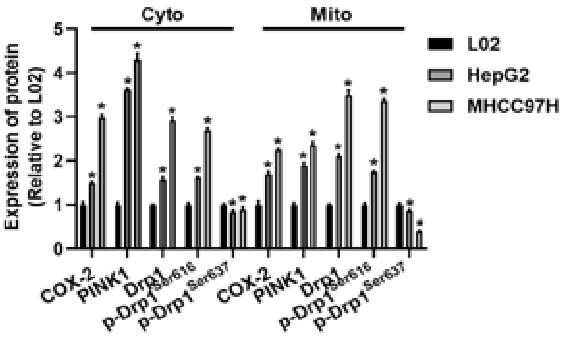

The corresponding protein quantification bar graphs for Fig.2H:

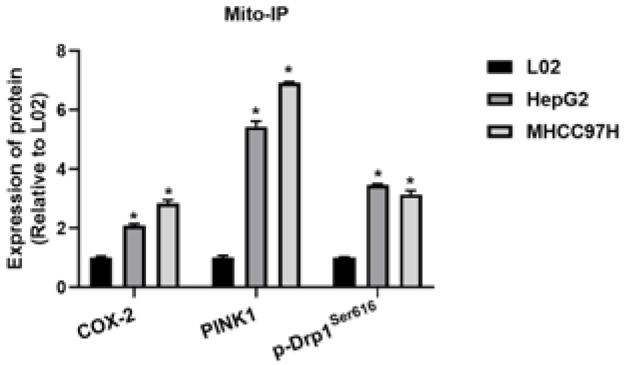

The corresponding protein quantification bar graphs for Fig.2I:

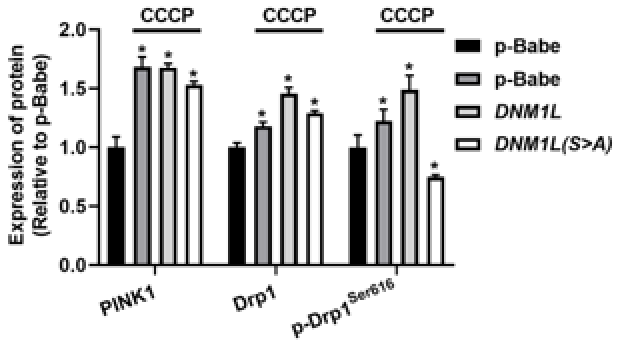

The corresponding protein quantification bar graphs for Fig.2J:

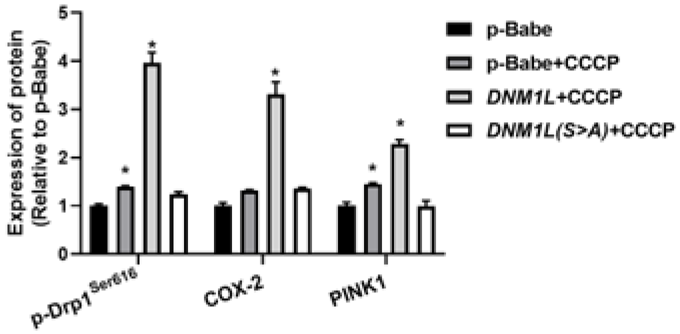

The corresponding protein quantification bar graphs for Fig.3A:

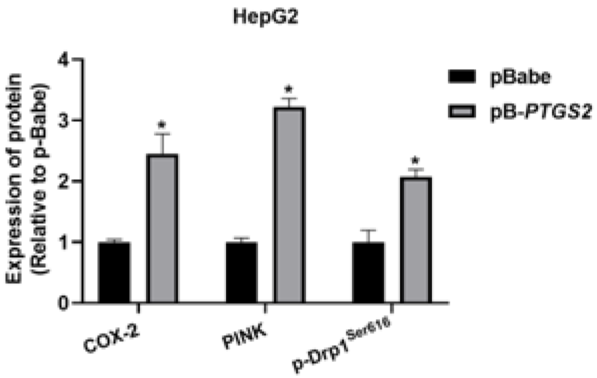

The corresponding protein quantification bar graphs for Fig.3B:

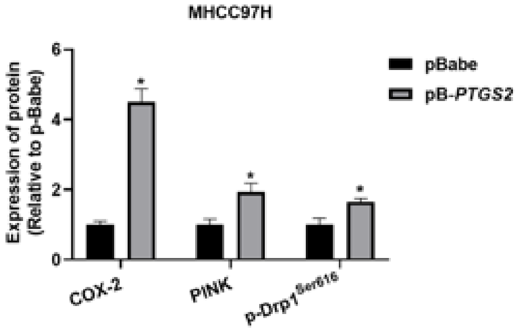

The corresponding protein quantification bar graphs for Fig.3D:

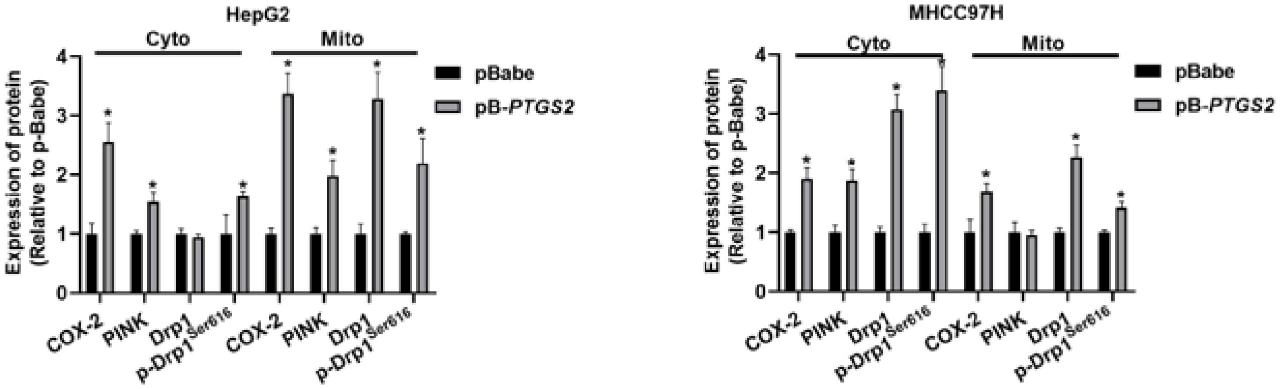

The corresponding protein quantification bar graphs for Fig.3G:

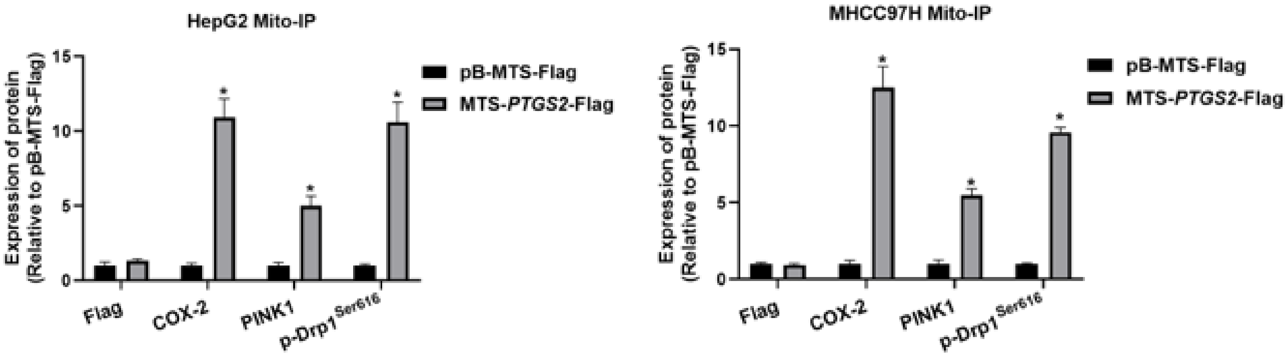

The corresponding protein quantification bar graphs for Fig.4C:

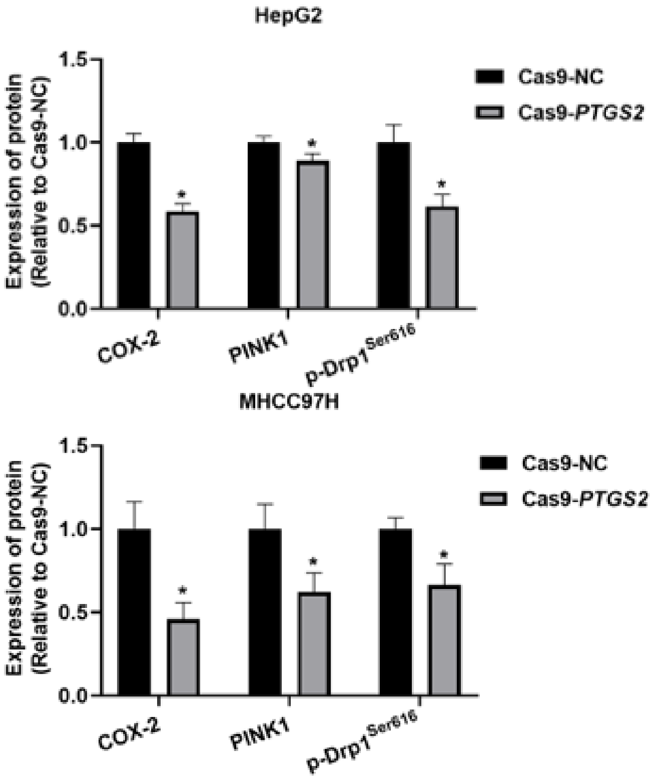

The corresponding protein quantification bar graphs for Fig.4E:

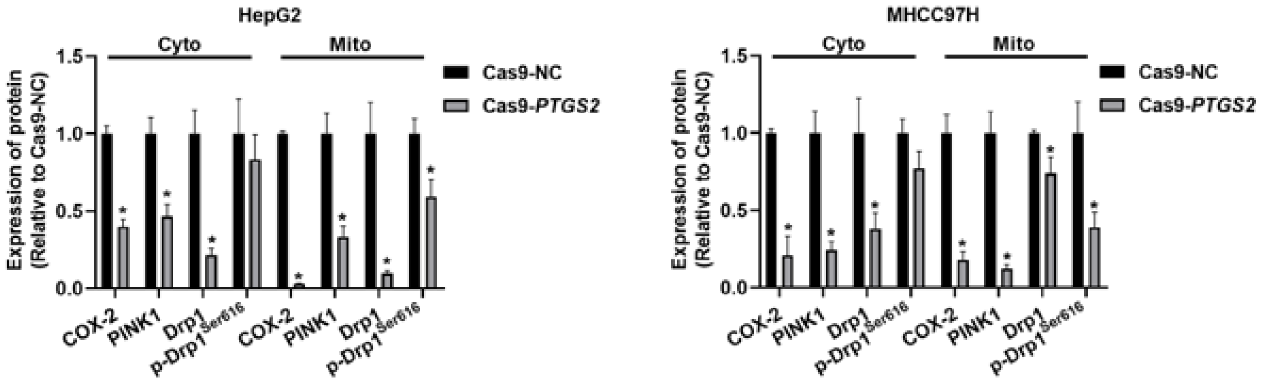

The corresponding protein quantification bar graphs for Fig.4G:

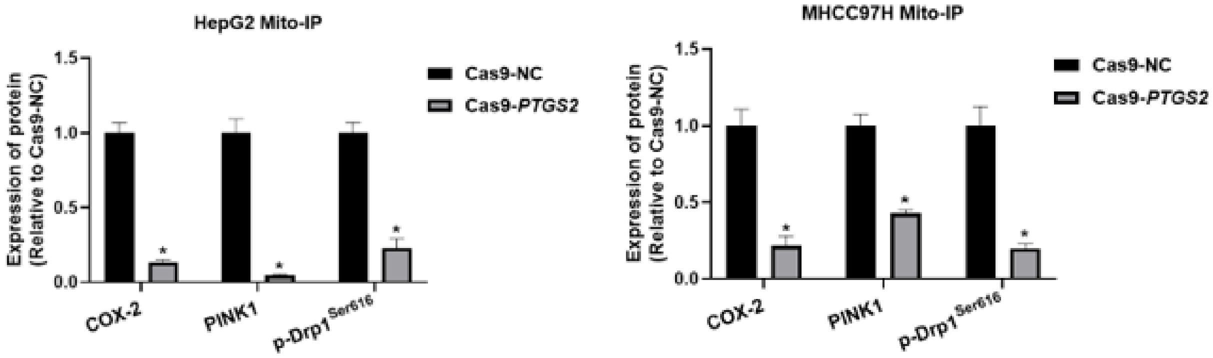

The corresponding protein quantification bar graphs for Fig.5E:

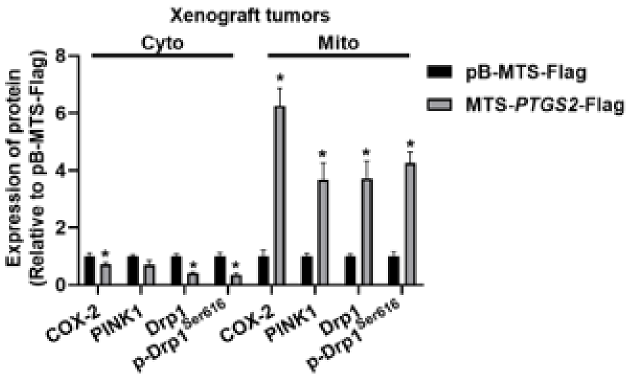

The corresponding protein quantification bar graphs for Fig.5F:

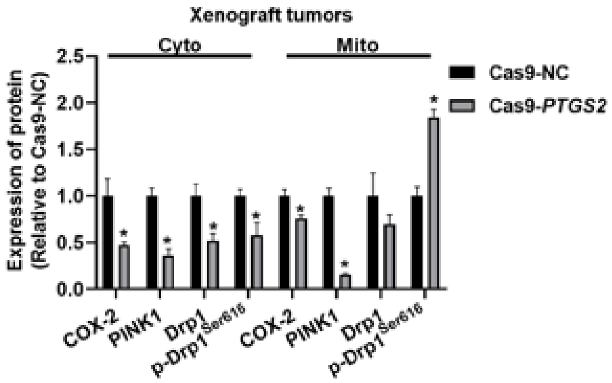

The corresponding protein quantification bar graphs for Fig.6A:

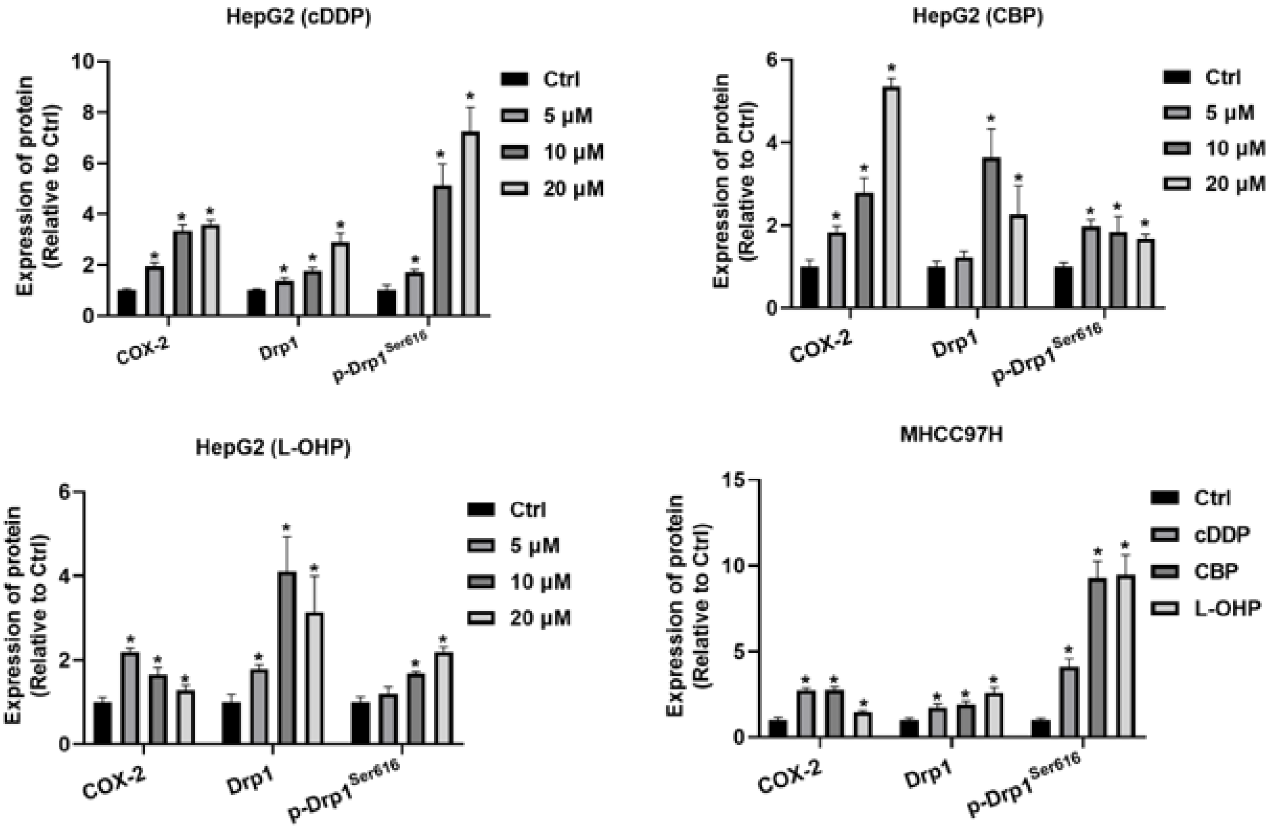

The corresponding protein quantification bar graphs for Fig.6D:

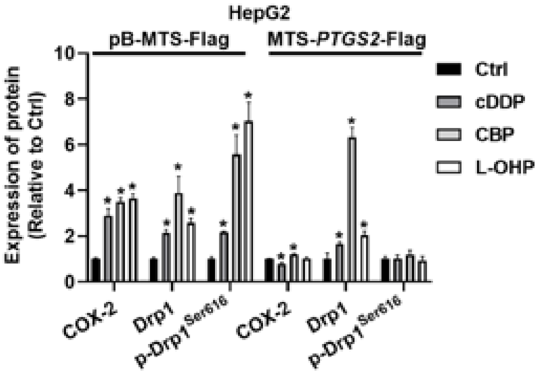

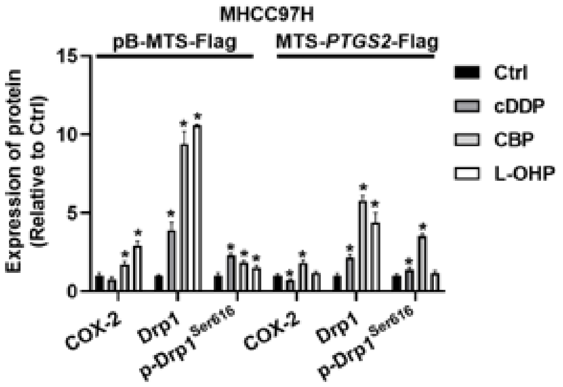

The corresponding protein quantification bar graphs for Fig.6F:

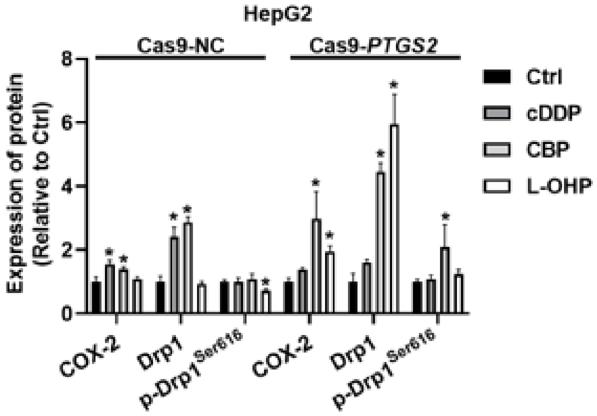

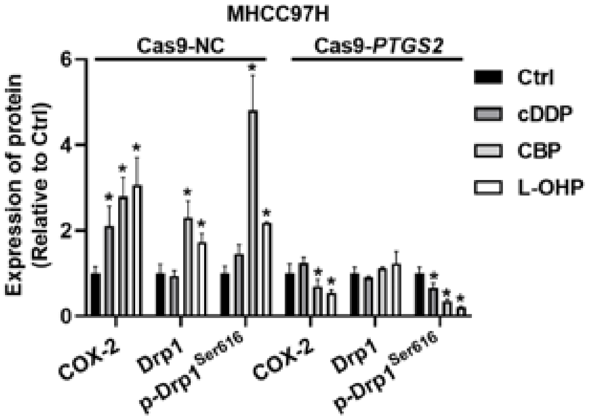

The corresponding protein quantification bar graphs for Fig.7A:

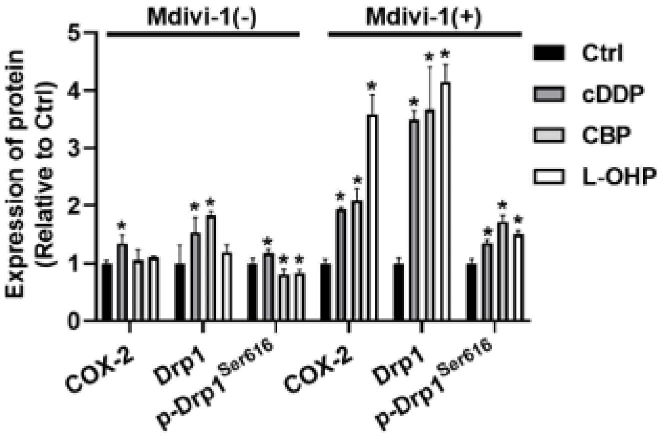

The corresponding protein quantification bar graphs for Fig.7E:

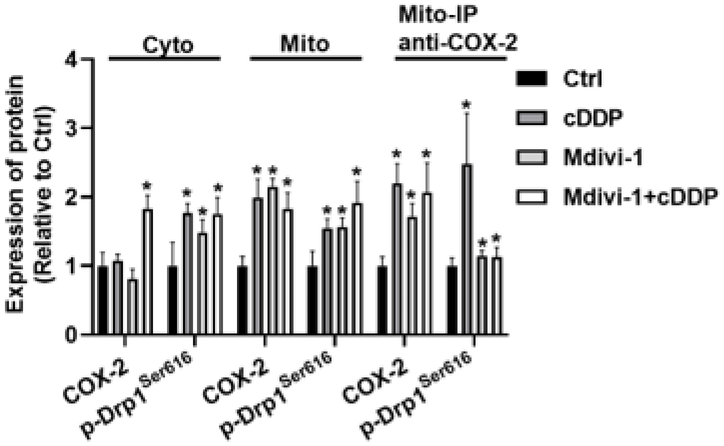

The corresponding protein quantification bar graphs for Fig.7F:

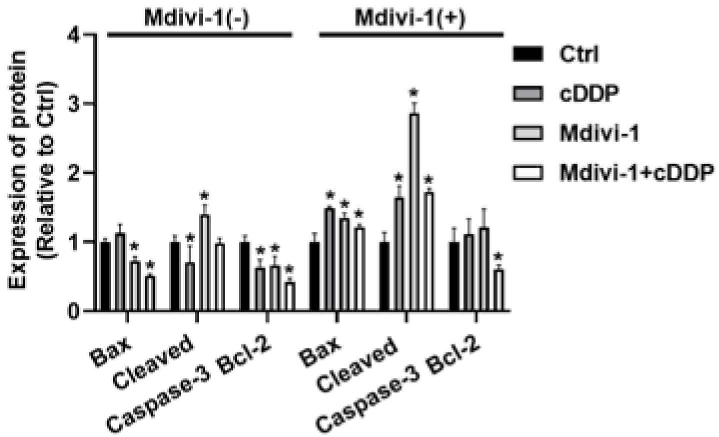

The corresponding protein quantification bar graphs for Fig.7H:

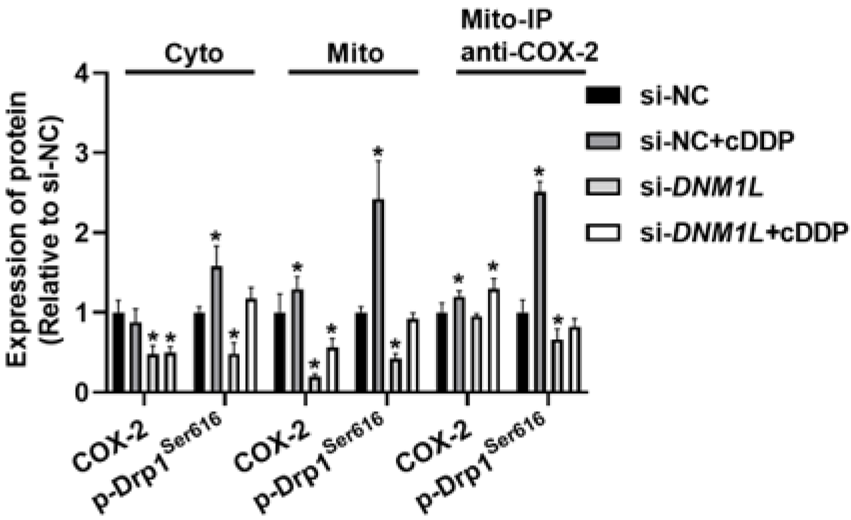

The corresponding protein quantification bar graphs for Fig.7I:

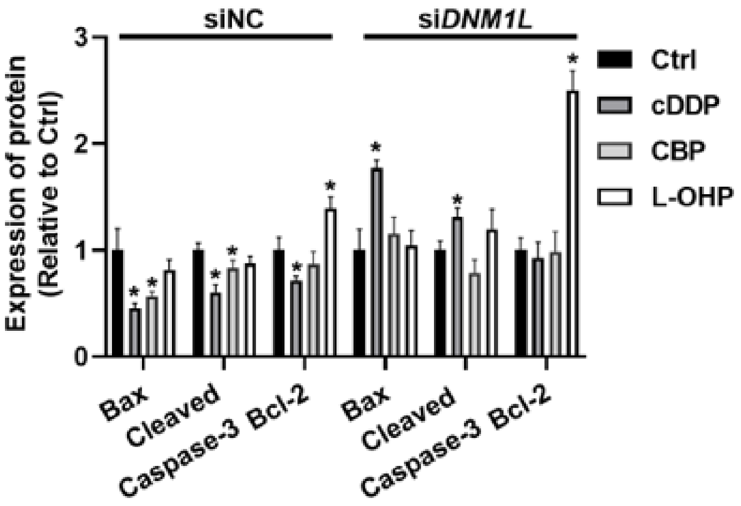

The corresponding protein quantification bar graphs for Fig.8B:

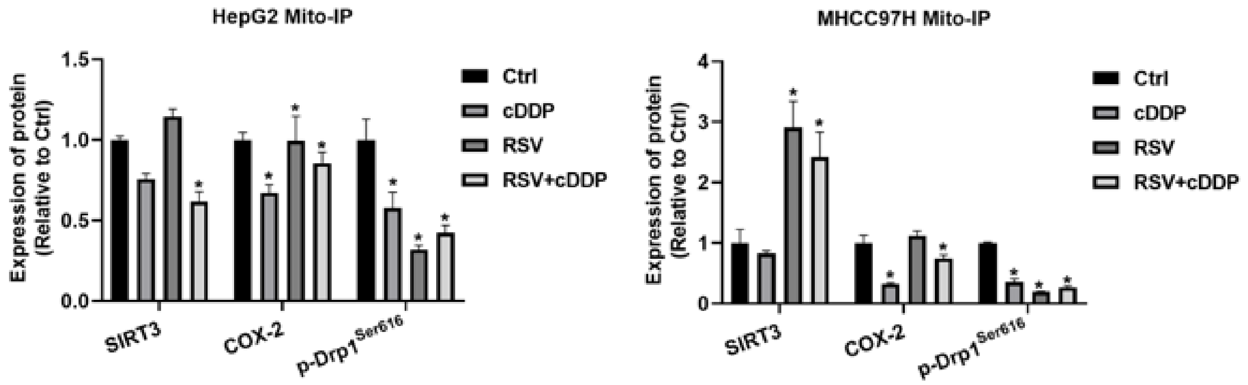

The corresponding protein quantification bar graphs for Fig.8C:

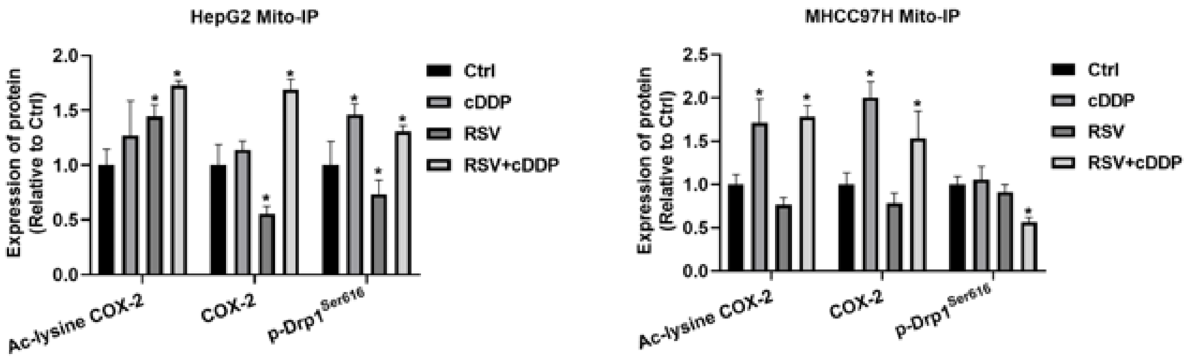

The corresponding protein quantification bar graphs for Fig.8D:

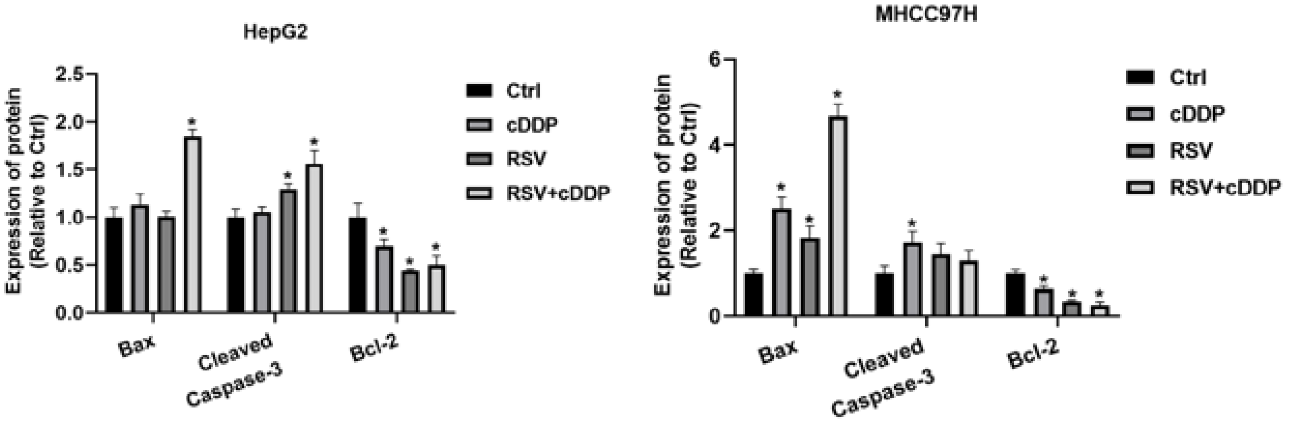

The corresponding protein quantification bar graphs for Fig.8F:

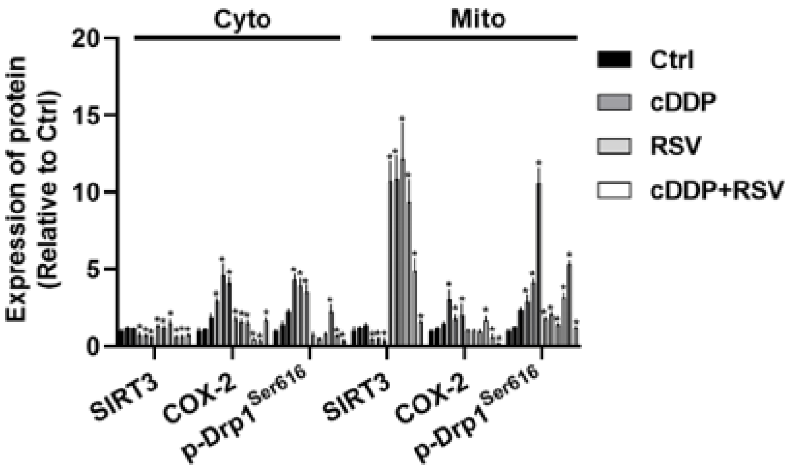

The corresponding protein quantification bar graphs for Fig.S3B:

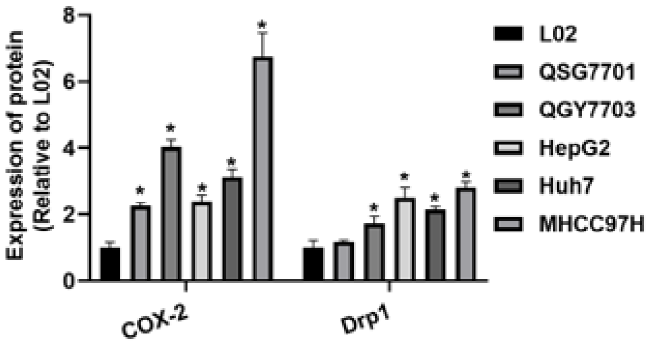

The corresponding protein quantification bar graphs for Fig.S4B:

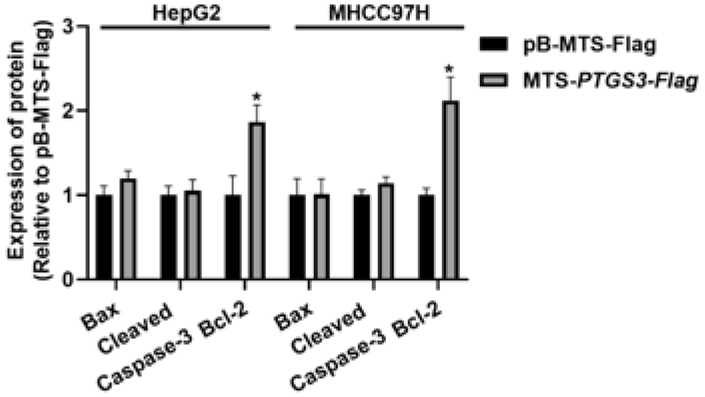

The corresponding protein quantification bar graphs for Fig.S5E:

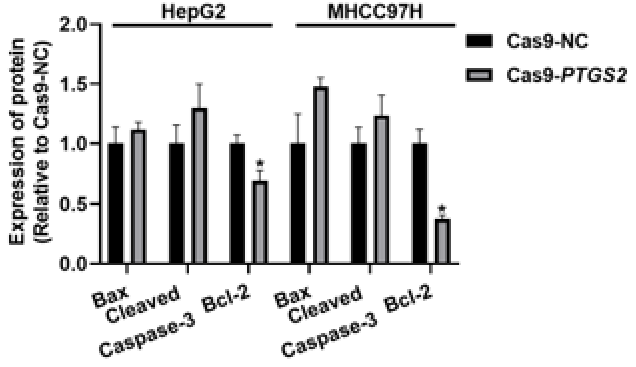

The corresponding protein quantification bar graphs for Fig.S7B:

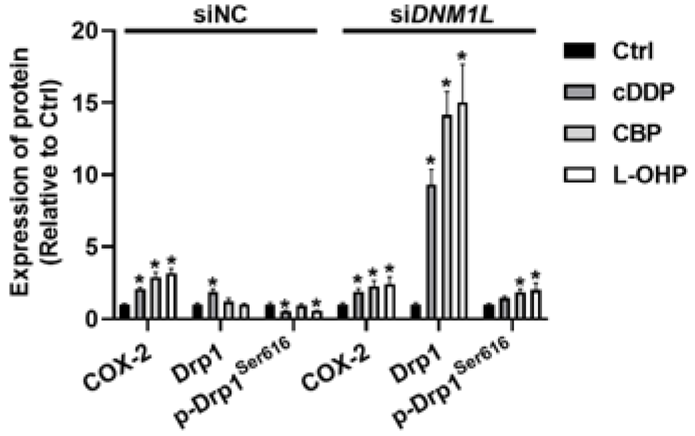

The corresponding protein quantification bar graphs for Fig.S8B:

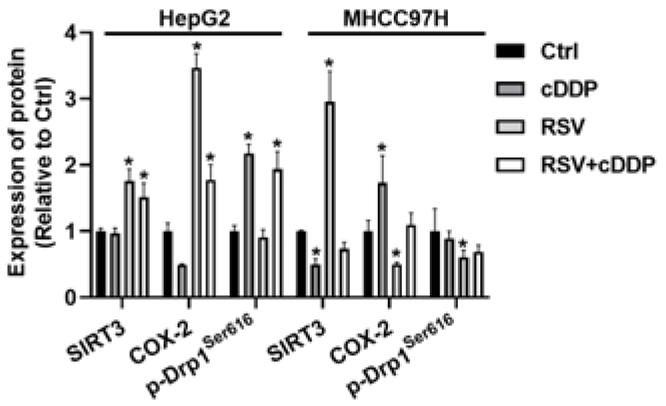

The corresponding protein quantification bar graphs for Fig.S8F:

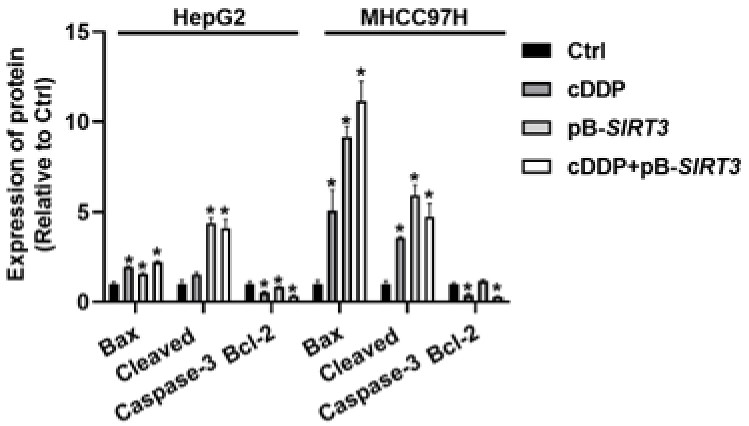

The corresponding protein quantification bar graphs for Fig.S8I:

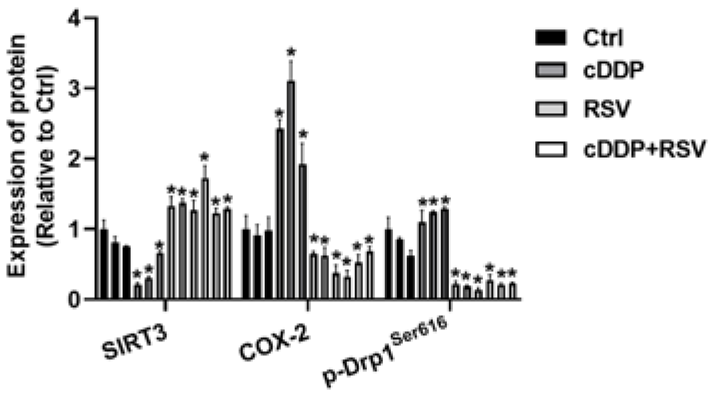

Figure S10. All western blots have been quantified and represented graphically with statistics (Mean ± SD).

Table S1. The GSE104310 and GSE36376 databases' detailed information is presented.

Data source	Platform	Sample No.	Organism	Source URL
GEO (GSE104310)	GPL16791 Illumina HiSeq 2500	20<br>Normal = 10<br>Tumor = 10	Homo sapiens	https://www.ncbi.nlm.nih.gov/geo/query/acc.cgi?acc=GSE104310
GEO (GSE36376)	GPL10558 Illumina HumanHT-12 V4.0 expression beadchip	433<br>Normal = 193<br>Tumor = 240	Homo sapiens	https://www.ncbi.nlm.nih.gov/geo/query/acc.cgi?acc=GSE36376
